# Rapidly progressive dementia with thalamic degeneration and peculiar cortical prion protein immunoreactivity, but absence of proteinase K resistant PrP: a new disease entity?

**DOI:** 10.1186/2051-5960-1-72

**Published:** 2013-11-11

**Authors:** Gabor G Kovacs, Alexander Peden, Serge Weis, Romana Höftberger, Anna S Berghoff, Helen Yull, Thomas Ströbel, Stefan Koppi, Regina Katzenschlager, Dieter Langenscheidt, Hamid Assar, Elisabeth Zaruba, Albrecht Gröner, Till Voigtländer, Gina Puska, Eva Hametner, Astrid Grams, Armin Muigg, Michael Knoflach, Lajos László, James W Ironside, Mark W Head, Herbert Budka

**Affiliations:** 1 Institute of Neurology, Medical University of Vienna and Austrian Reference Centre for Human Prion Diseases Vienna, Vienna, Austria; 2 National CJD Research & Surveillance Unit, University of Edinburgh, Edinburgh, UK; 3 Laboratory of Neuropathology, Department of Pathology and Neuropathology, State Neuropsychiatric Hospital Wagner-Jauregg, Linz, Austria; 4 Department of Neurology, Landeskrankenhaus Rankweil, Rankweil, Austria; 5 Department of Neurology and Karl Landsteiner Institute for Neuroimmunological and Neurodegenerative Diseases, Donauspital / Danube Hospital, Vienna, Austria; 6 Neuromedizinische Ambulanzzentrum, State Neuropsychiatric Hospital Wagner-Jauregg, Linz, Austria; 7 2nd Department of Neurology, Hietzing Hospital and Neurological Center Rosenhügel, Vienna, Austria; 8 CSL Behring, Marburg, Germany; 9 Department of Anatomy, Cell and Developmental Biology, Eötvös Lóránd University of Sciences, Budapest, Hungary; 10 Department of Neurology, University Clinics Innsbruck, Innsbruck, Austria; 11 Department of Neuroradiology, University Clinics Innsbruck, Innsbruck, Austria; 12 Institute of Neuropathology, University Hospital Zürich, Zürich, Switzerland

**Keywords:** Conformational assay, Prion protein, Protease-sensitive PrP^Sc^, Prionopathy, Thalamic degeneration

## Abstract

**Background:**

Human prion diseases are a group of rare fatal neurodegenerative conditions with well-developed clinical and neuropathological diagnostic criteria. Recent observations have expanded the spectrum of prion diseases beyond the classically recognized forms.

**Results:**

In the present study we report six patients with a novel, apparently sporadic disease characterised by thalamic degeneration and rapidly progressive dementia (duration of illness 2–12 months; age at death: 55–81 years). Light and electron microscopic immunostaining for the prion protein (PrP) revealed a peculiar intraneuritic distribution in neocortical regions. Proteinase K resistant PrP (PrP^res^) was undetectable by Western blotting in frontal cortex from the three cases with frozen tissue, even after enrichment for PrP^res^ by centrifugation or by phosphotungstic acid precipitation. Conformation-dependent immunoassay analysis using a range of PK digestion conditions (and no PK digestion) produced only very limited evidence of meaningful D-N (denatured/native) values, indicative of the presence of disease-associated PrP (PrP^Sc^) in these cases, when the results were compared with appropriate negative control groups.

**Conclusions:**

Our observation expands the spectrum of conditions associated with rapidly progressive dementia and may have implications for the understanding of the pathogenesis of prion diseases.

## Background

Human prion diseases (PrDs) are a group of rare fatal neurodegenerative conditions with well-developed clinical and neuropathological diagnostic criteria. The current consensus view of their underlying pathological process largely focuses on the presence of a pathological, disease-associated prion protein (PrP) conformer (PrP^Sc^) that is involved in neurotoxicity, infectivity and strain differentiation [1]. PrP^Sc^ is distinguished from the physiological cellular conformer of PrP (PrP^C^) by an increased β-pleated sheet structure [2], and relative resistance to digestion with proteinase K (PK) (the products that are resistant to PK-digestion are termed PrP^res^). In human PrDs three major PK-resistant PrP types have been described that are readily identifiable by immunoblotting with a range of anti-PrP antibodies: type 1 (21 kDa non-glycosylated PrP^res^), type 2 (19 kDa non-glycosylated PrP^res^), and lower molecular mass (approximately 8 kDa) fragments [3,4]. These PrP^res^ types are thought to represent different conformational states of the protein. Together with the codon 129 polymorphism (methionine/valine, M/V), types 1 and 2 are important for the molecular classification of sporadic PrDs [5].

A different approach to the detection of PrP^Sc^ is employed in the conformation-dependent immunoassay (CDI). This uses increasing concentrations of the chaotropic salt guanidine hydrochloride (GdnHCl) to unmask epitopes in PrP that become hidden during the formation of PrP^Sc^. This method has provided evidence that PrP^Sc^ also exists in abundance in protease-sensitive forms (senPrP^Sc^) in human PrDs [6,7]. However, CDI is not yet used routinely in human PrD diagnosis.

According to the etiological classification of PrDs, sporadic (idiopathic) Creutzfeldt-Jakob disease (sCJD) is the most frequently occurring form. This is a fatal disorder characterised neuropathologically by widespread spongiform change in the brain and prominent deposition of the disease-associated PrP with distinct morphologies [8]. However, one molecular form of sCJD differs in that only subtle spongiform change is seen and that prominent degeneration in the thalamus and inferior olives is the most prominent feature [9]. Indeed, this form was termed the thalamic form of CJD or sporadic fatal insomnia (sFI), due to its close phenotypic and biochemical similarity to a genetic PrD, fatal familial insomnia (FFI), which is associated with the D178N mutation in the prion protein gene (*PRNP*) and methionine encoded in the mutated allele at codon 129 [9]. Importantly, PrP^res^ has been found (type 2) in both sFI and FFI, and immunostaining for PrP reveals mild, but distinct synaptic type immunodeposits [5]. Sporadic FI has always been associated with methionine homozygosity at codon 129 (and sometimes termed “MM type 2 thalamic CJD”) [5].

Recent observations have expanded the spectrum of PrDs beyond the classically recognized forms associated with abundant PrP^res^ to include a disease called variably protease-sensitive prionopathy (VPSPr) [10]. Immunoblotting shows a ladder-like pattern of protease-resistant PrP fragments, extending from approximately 8 kDa to the 18–30 kDa molecular mass range. Detection of the poorly protease-resistant PrP^Sc^ in VPSPr can require particular antibodies such as 1E4 (rather than by the 3F4 anti-PrP antibody) or enrichment after PK treatment [10,11] for its detection by Western blotting. Nevertheless, in VPSPr, spongiform change and the widespread presence of disease-associated PrP immunoreactivity are observed, in conjunction with PrP^res^ that is detectable by immunoblotting. Thus, in PrDs the disease-associated PrP^Sc^ may be in the form of PrP^res^ or PrP^sen^ (i.e. protease-sensitive PrP^Sc^ or senPrP^Sc^) but is distinguishable from the physiological PrP^C^. The roles of and relationship between senPrP^Sc^ and PrP^res^ are not yet known, however no PrDs have yet been described in which senPrP^Sc^ is detectable in the apparent absence of PrP^res^.

In the present study we report six individuals who presented clinically with rapidly progressive dementia and neurological symptoms. Neuropathological examination did not reveal spongiform encephalopathy, but thalamic degeneration and a peculiar PrP immunoreactivity was found in the cortex. The identification of such cases expands the spectrum of conditions associated with rapidly progressive dementia and may have implications for the understanding of the pathogenesis of prion diseases.

## Methods

### Selection of cases

The patients were encountered and systematically investigated as part of the surveillance for human PrDs in Austria or referred to the Institute of Neurology, Medical University Vienna, for neuropathological autopsy diagnosis. Samples were collected following local regulations as part of the study (“Molecular neuropathologic investigation of neurodegenerative diseases”) approved by the Ethical Committee of the Medical University of Vienna following the principles of the Helsinki declaration. Clinical and neuroradiological data were obtained retrospectively. Genetic analysis was performed using genomic DNA isolated from blood samples or frozen brain tissue as published previously [12]. For comparative purposes we selected one Austrian FFI case (D178N-129MV), and two UK cases of sporadic CJD (one of the common MM1 subtype and one of the rare MM2T or sFI subtype), which were obtained from the UK MRC Edinburgh Brain Banks (CJD Brain and Tissue Bank, LREC 2000/4/175, Prof J.W. Ironside). FFI and sFI cases were selected because they also show prominent thalamic degeneration, variable amounts of PrP immunoreactivity and PrP^res^. The brain from each of these cases used as positive controls had previously been examined histologically and biochemically, and a definite diagnosis of sporadic CJD or sFI/FFI had been reached by established criteria [13,14]. The protease-resistant PrP (PrP^res^) isotype found in CJD brains was classified as type 1, 2A, or 2B as previously described according to the nomenclature of Parchi and colleagues [3,5,15]. The polymorphic status of codon 129 of the *PRNP* of each UK case was determined by restriction fragment length polymorphism [16]. The presence of the D178N mutation in the cases of FFI and the absence of prion disease-associated mutations in the sFI and the sCJD cases were determined by sequencing of the *PRNP* gene ORF. Cerebral cortex samples from ten cases from the Medical Research Council Edinburgh Brain and Tissue bank were analysed by CDI as controls. Five of these were sudden death cases with no history of a neurological condition (designated ‘sudden death’), whereas another five were cases initially referred to NCJDRSU as suspected CJD cases, but given an alternative final diagnosis (designated ‘non-CJD’). The non-CJD cases had pathological diagnoses of Alzheimer’s disease (n = 3), frontotemporal lobar degeneration motor neurone disease and cerebral infarction. The VPSPr brain tissue sample used in this study was from a patient who was VV at codon 129 of the *PRNP* gene and was a generous gift from Dr. Gambetti (National Prion Disease Pathology Reference Centre, USA).

### Neuropathology

Formalin fixed, paraffin-embedded tissue blocks from the investigated cases were evaluated including the following anatomical regions: frontal, anterior cingulate, parietal, temporal, occipital cortex, hippocampus, entorhinal cortex, amygdala, basal ganglia, thalamus, mesencephalon, pons, medulla oblongata, and cerebellum. In addition to Haematoxylin and Eosin staining, the following monoclonal (mouse) antibodies were used for immunohistochemistry: anti-PrP 3F4 (epitope: aa. 106–112; 1:1,000, Signet/Covance, Berkeley, CA, USA), anti-PrP L42 (epitope: aa. 141–159; 1:300, FRC for Virus Diseases of Animals, Dr. M.H. Groschup, Tübingen, Germany) [17], anti-PrP 12F10 (epitope: aa. 142–160; 1:1,000, Cayman Chemical, Ann Arbor, MI, USA), anti-PrP BG4 (epitope: aa. 23–85), anti-PrP KG9 (epitope: aa. 140–180) (both 1:1,000, TSE Resource Centre, Roslin Institute, Edinburgh, U.K.), anti-PrP 6H4 (epitope: aa. 144–152; 1:500, Prionics, Schlieren, Switzerland), monoclonal anti-p62 (1:1,000, BD Transduction, Lexington KY, USA), monoclonal anti-ubiquitin (1:50,000, Millipore, Temecula, CA, USA), anti-tau AT8 (pS202/pT205, 1:200, Pierce Biotechnology, Rockford, IL, USA), anti-phospho-TDP-43 (pS409/410, 1:2,000, Cosmo Bio, Tokyo, Japan), anti-α-synuclein (1:2,000, clone 5G4, Roboscreen, Leipzig, Germany; specific for disease-associated form) [18], anti-Aβ (1:50, clone 6 F/3D, Dako, Glostrup, Denmark), and anti-amyloid precursor protein, APP (1:500, Millipore, Billerica, MA, USA). Furthermore polyclonal (rabbit) anti-FUS (1:1,000, SIGMA, Saint Louis, MO, USA) and anti-GFAP (1:5,000, Dako, Glostrup, Denmark) antibodies were also applied. The DAKO EnVision© detection kit, peroxidase/DAB, rabbit/mouse (Dako, Glostrup, Denmark) was used for visualization of antibody reactions. For the purpose of surveillance for PrDs in Austria, since January 2002 we have used the antibody 12F10 for immunohistochemical analyses of brain sections. Between January, 2002 and January, 2013 (11 years), we have systematically evaluated 312 brains that were processed and immunostained using the same protocol described here. In addition to 170 definitive CJD cases, 3 cases with FFI (two D178N M129M and one M129V), 2 cases with Gerstmann-Sträussler-Scheinker disease (GSS P102L), single cases with 144 base or 120 pair insertion, a total of 135 brains were received for examination as suspect prion disease but were found to have other disorders, and prion disease was formally excluded. This cohort comprises diagnoses of Alzheimer’s disease, Lewy body dementia, frontotemporal lobar degeneration with TDP-43 proteinopathy, corticobasal degeneration, progressive supranuclear palsy, encephalitis, vascular lesions (including the thalamus), carcinomatous meningitis, metastases, metabolic and hypoxic/ischemic encephalopathy. In addition we evaluated 8 cases with pathology in the thalamus (six Wernicke encephalopathy; age range: 34–73 years at death, 4 men; and two with paramedian infarct, 78 and 86 years-old women).

In this study, neuropathological alterations (spongiform change, astrogliosis/neuronal loss, degree of various protein depositions) were semi-quantitatively (none, mild, moderate, severe) evaluated in different anatomical regions. For PrP immunohistochemistry we compared three pre-treatment protocols: sections were treated (1) in autoclave (121°C in distilled water) followed by 5 minutes in 98% formic acid, (2) by additional treatment with PK (10 μg/ml, Dako PK Ready-to-use, Code S3020), or (3) only 20 minutes citrate buffer (pH 6) pre-treatment in a steamer (95°C).

### Paraffin-embedded tissue (PET) blotting

This was performed with slight modifications according to published protocols [19]. Briefly, sections of formalin fixed, paraffin embedded brain tissue were placed onto a nitrocellulose membrane. Sections were treated with 250 μg/ml PK or with 150 μg/ml thermolysin (both Sigma-Aldrich) for eight hours at 56°C. Thermolysin was used because it has been shown to degrade PrP^C^ while preserving both PK-sensitive and PK-resistant isoforms of disease-associated PrP [20]. After washing, we treated the membranes with 3 M guanidine isothiocyanate for 10 minutes. For immunodetection we used the antibody 3F4 (1:500).

### Immunogold electron microscopy

After incubation of the deparaffinised tissue sections with anti-PrP (12F10) antibody, we applied ultrasmall gold conjugated secondary antibody (Aurion, Wageningen, The Netherlands) followed by silver-enhancing method. Slides were mounted with resin and examined by light microscopy. Selected areas containing immunoreactive structures were re-embedded for ultrathin sectioning [21]. We used a JEOL-1011 electron microscope to analyse the ultrastructural localization of PrP-specific immunolabeling in the ultrathin sections.

### Immunoblotting

#### Analysis of brain samples by Western blotting, including centrifugal concentration

For Western blot analysis, samples were homogenized in nine volumes (weight to volume, w/v) of tris-buffered saline, pH 7.6, containing 0.5% Nonidet P40 and 0.5% sodium deoxycholate. Aliquots of the cleared 10% brain homogenates were subjected to limited proteolysis by digestion with PK at 50 μg/ml for one hour at 37°C. The reaction was terminated by addition of Pefabloc SC (Roche, Burgess Hill, UK) to a final concentration of 1 mM. PK-treated samples were either analysed by Western blotting directly, or alternatively 100 or 200 μl samples were centrifuged at 20,800 g for one hour at 4°C and the pellets were analysed. Polyacrylamide gel electrophoresis and Western blotting was performed using the NuPAGE Novex gel system (Invitrogen, Paisley, UK) as described previously [22]. The immunodetection of PrP was achieved using the monoclonal antibodies 3F4 (Millipore, Watford, UK), 1E4 and 94B4 (both provided by Dr Jan Langeveld [Central Veterinary Institute of Wageningen UR, Lelystad, The Netherlands]). The antibodies were used at final concentrations of 75 ng/ml, 1 μg/ml and 500 ng/ml respectively for one hour.

#### Analysis of brain samples by Western blotting after sodium phosphotungstic acid precipitation

Sodium phosphotungstic acid (NaPTA) precipitation was performed as published previously [23,24], with a few minor modifications. Briefly, 0.5 ml samples of 10% (wt/vol) cleared brain homogenates prepared for CDI analysis in 2% sarkosyl/PBS (see below) were diluted with a further 0.5 ml of 2% sarkosyl/PBS and incubated for 10 minutes at 37°C. Benzonase (Sigma E1014) and MgCl_2_ were added at final concentrations of 50 U/ml and 1 mM, respectively, and incubation at 37°C was continued for a further 30 minutes. 81 μl of a stock solution of 4% (wt/vol) NaPTA and 34 mM MgCl_2_, pH 7.4, was added (final concentration of NaPTA, 0.3% [wt/vol]), and precipitation was allowed to occur for 30 minutes at 37°C. The samples were centrifuged at 20,800 *g* for 30 minutes at 37°C. The pellets were resuspended in 20 μl of 0.1% (wt/vol) sarkosyl in PBS, pH 7.4, and digested with 50 μg/ml PK for 30 minutes at 37°C. Digestion was terminated by the addition of 1 mM Pefabloc SC (Roche, Burgess Hill, UK). Electrophoresis and transfer was performed as described above but the detection reagent used was SuperSignal West Femto maximum sensitivity substrate (Pierce, Rockford, IL, USA). Images were again obtained using the ChemiDoc™ XRS system and Image Lab software (Bio-Rad).

### Conformation-dependent immunoassay (CDI)

#### Homogenization of brain samples for CDI analysis

Frozen tissue samples were weighed and homogenised in phosphate buffered saline (P5493, Sigma-Aldrich, Dorset, UK) containing 2% sarkosyl (N-laurylsarcosinate 61745, Sigma-Aldrich) using a buffer volume that gave a final tissue concentration of 10% (wt/vol). The homogenization was performed in tubes containing lysing matrix D using the Fast-Prep instrument (MP Biomedicals, Solon, Ohio, USA). Three 45-second rounds of lysis were performed at 6.5 ms^-1^ separated by five-minute intervals to allow cooling. The homogenates were then cleared of cellular debris by centrifugation at 5200 *g* for five minutes at 4°C. The supernatants were transferred to fresh tubes and stored at −80°C.

#### Analysis of brain homogenates by CDI

Brain homogenates were either assayed by CDI directly, or following limited proteolytic digestion with 2.5 or 50 μg/ml PK for one hour at 37°C. PK digestion was terminated by the addition of 1 mM Pefabloc SC. The CDI method used in this study was performed as described previously [7,25] (but omitting the NaPTA precipitation step). In CDI PrP^Sc^ is detected on the basis of an increase in signal following sample denaturation as epitopes hidden within misfolded PrP^Sc^ become exposed. In CDI, PrP is detected using the dissociation enhanced lanthanide fluorescence immunoassay (DELFIA) technology of PerkinElmer (PerkinElmer, Cambridge, UK).

Samples of brain homogenates prepared as described above were divided into two equal aliquots. One aliquot was mixed with an equal volume of 8 M guanidine hydrochloride (GdnHCl) and denatured (D) by incubating at 81°C for six minutes and the other was diluted without GdnHCl and left at room temperature and designated native (N). Both D and N samples were adjusted to a final volume of 650 μl and a final guanidine concentration of 308 mM, with water containing complete™ EDTA-free protease inhibitors (Roche). A 96-well black polystyrene plate (Fisher, Loughborough, UK) was coated with 2.5 μg/ml (0.5 μg/well) anti-PrP capture antibody MAR-1 (CSL Behring, Marburg, Germany) diluted in carbonate-bicarbonate buffer overnight at room temperature. The wells were washed four times with DELFIA wash buffer (PerkinElmer) using a Model 1575 Immunowash microplate washer (Bio-Rad). After saturating the plate by shaking for one hour with 0.5% (wt/vol) bovine serum albumin and 6% D-sorbitol (wt/vol) in DELFIA wash buffer, the wells were washed four times and the D or N samples were loaded in triplicate onto the plate (200 μl/well).

The samples were incubated on the plate for two hours at room temperature with shaking. The plate was washed four times and europium-conjugated PrP antibody 3F4 (~25 ng/ml) in DELFIA assay buffer (200 μl/well) was added to all wells and incubated for two hours at room temperature with shaking. The wells were then washed six times and DELFIA enhancement solution (200 μl/well) was added to all wells. After incubation for five minutes at room temperature with shaking, the time-resolved fluorescence signals for the denatured and native samples were measured using a Victor 2 fluorometer (PerkinElmer).

#### Calibration of CDI

The CDI assay for PrP was calibrated using full-length human recombinant PrP (recHuPrP, amino acids 23–231, methionine at codon 129) purified as described previously [26]. A range of dilutions from 40 μg/ml to 10 μg/ml of this recHuPrP was prepared using PBS containing 2% sarkosyl. Twenty-five μl samples of these dilutions were mixed with an equal volume of 8 M GdnHCl and denatured as described above. These samples were adjusted to 650 μl, with water containing complete™ EDTA-free protease inhibitors and analysed as described above. The time resolved fluorescence counts obtained for the reHuPrP dilution series were used to plot a standard curve of counts versus micrograms of PrP. This curve was used to convert the CDI signals obtained for known volumes of 10% (wt/vol) brain homogenates into values with units of micrograms of PrP per gram of brain tissue. The mean values obtained for the N and D samples are assumed to correspond to PrP^C^ and total PrP (PrP^C^ + PrP^Sc^) respectively. The mean D-N value for each sample tested was used as an estimate of PrP^Sc^.

## Results

### Case reports

#### Case 1

A 77-year-old woman presented with progressive cognitive decline associated with psychiatric symptoms. Later gait ataxia appeared and myoclonus was observed in the extremities. Pyramidal signs were observed terminally. Examination of CSF 14-3-3 revealed strong positivity, with a normal cell count and moderately elevated protein (64 mg/dl), while the EEG did not show periodic sharp waves or triphasic components. MRI was not performed. She died after a 12-month illness in April 2010. Clinically sCJD was suspected. No mutations were found in the *PRNP* gene; the codon 129 was MM.

#### Case 2

A 55-year-old woman presented with progressive dementia and a gait disorder associated with choreiform movements developing within a few months. She received tiapridhydrochloride and haloperidol. Screening for *Huntingtin* mutations excluded any alteration. In January 2011 she was admitted to hospital due to impairment in gait and the appearance of myoclonus. An akinetic-rigid state was observed and she was unable to walk. Cranial CT revealed widening of the sulci in the frontotemporal areas and moderate ventricle enlargement. Routine laboratory parameters were in a normal range. After admission, a generalized epileptic seizure occurred, and levetiracetam therapy was initiated with discontinuation of the neuroleptic drugs. Only mild chorea was noted at this time. EEG revealed theta-delta waves with focal spikes in the temporal region bilaterally. Mini Mental State was 10/30. Soon dysphagia appeared with recurrent aspiration pneumonia. After a temporary stay at home, she was re-admitted in late April 2011. Pyramidal signs and akinetic mutism were observed and she died due to respiratory insufficiency after 12 months of illness. CJD was suspected only terminally, and CSF 14-3-3 and MRI were not conducted. No mutations were found in the *PRNP* gene; the codon 129 was MM.

#### Case 3

This 81-year-old man presented with visual hallucinations, which rapidly progressed to cortical blindness. In addition ataxic gait and progressive cognitive decline were documented. Later myoclonus was observed in the extremities. Examination of CSF 14-3-3 revealed moderate positivity, with a normal cell count, while the EEG did not show periodic sharp waves or triphasic components. Cranial MRI did not reveal signal alterations in the thalamus, basal ganglia or cortex (Additional file 1: Figure S1a). He died after a 2-month illness in April 2013. Clinically sCJD was suspected. No mutations were found in the *PRNP* gene; the codon 129 was MM.

#### Case 4

This 74-year-old woman with a 6-month history of ataxia and cognitive decline was admitted to a Neurology Department. Her father had died at the age of 63, and her mother at the age of 73 years, both with pulmonary disease, but without a neuropsychiatric disorder. Neurological examination revealed mild rigidity of the neck muscles and mild gaze impairment in all directions, while myokymia was observed in the temporal and masseter muscles. In addition hypacusis, hypomimia, myoclonus in the shoulder muscles, dysphagia, limb (right > left) and axial rigidity, ataxia, dysdiadochokinesia, slow psychomotor activity, acalculia, apraxia, and urinary incontinence were also described. Mini Mental State was 20/30, which decreased to 7/30 after a week. Repeated cranial MRI revealed ventricular enlargement with periventricular white matter T2 hyperintensities and moderate diffuse cortical atrophy (Additional file 1: Figure S1b). In addition, a tumor (neuroradiologically compatible with acoustic schwannoma) was observed in the left cerebellopontine angle (27x16x19 mm); this did not show progression. Increased pressure of CSF was not detected, and no clinical improvement was observed after taking 30 ml CSF. At this time urinary incontinence was not present. Examination of the CSF showed normal cell count. Protein 14-3-3 was moderately positive. Dopamine transporter (DAT)-SPECT showed loss of DAT density in the basal ganglia (left predominant) and was suggestive of Parkinson’s disease. However L-Dopa substitution did not lead to improvement. EEG was performed on days 4, 19, 31 and 62 after admission showing slow activity with occasional triphasic waves in the frontotemporal regions bilaterally (Additional file 1: Figure S1c). Soon after akinetic mutism developed and the patient died due to aspiration pneumonia after 6 months duration of illness in April 2011. Clinically sCJD was suspected. No mutations were found in the *PRNP* gene; the codon 129 was heterozygous (MV).

#### Case 5

This 62-year-old man was admitted to the Neurology department in July 2010, due to progressive gait disorder in the past 6 months. There was neither a history of neuropsychiatric disease in his parents nor a relevant personal medical history, except for alcohol abuse. Neurological examination revealed hypomimia, mild dysarthia, bradykinesia, limb ataxia (left > right) and distal hypesthesia in the lower limbs. Barthel index was 90/100, Mini Mental State 24/30, clock test 3/7. Cranial MRI showed diffuse cortical atrophy and DAT SPECT revealed bilateral decreased striatal expression. A neurodegenerative Parkinsonian syndrome, cognitive decline, alcohol abuse with hepatopathy (elevated plasma ASAT/GOT) and polyneuropathy were diagnosed; L-Dopa, low-dose dopamine-agonist, gabapentin, rasagilin, and dexibuprofen therapy was initiated. He was re-admitted to the hospital in January 2011 due to non-compliance with the therapy, depressive mood, visual hallucinations, agitation and ongoing alcohol abuse. CSF protein 14-3-3 was not assessed. He died due to pneumonia within 2 weeks. The duration of illness was 9 months. Dementia with Lewy bodies (DLB) was suspected clinically although it was felt that the patient’s long-standing alcohol abuse precluded a clinical diagnosis of probable DLB. Analysis of the *PRNP* gene was not performed.

#### Case 6

In March 2012 a 79-year-old woman with a 6-month history of progressive gait disorder, cognitive decline and most recently, change of character and oculomotor disturbance, was transferred to the clinic for further evaluation. The neurologic exam showed hypersomnolent apathy, amnesic syndrome, and impaired gaze. Cerebral MRI revealed bi-thalamic signal-alterations in FLAIR and diffusion-weighted sequences (Additional file 1: Figure S1d,e). EEG showed continuous generalized delta-theta activity but no periodic or triphasic waves. Extended CSF examination showed no signs of encephalitis or paraneoplastic disease. Due to the clinical presentation as well as matching imaging and laboratory findings with marked generalized vitamin and protein deficiency and a history of severe chronic gastritis, non-alcoholic Wernicke encephalopathy was initially suspected. Despite vitamin and protein replacement therapy the patient’s clinical status deteriorated and the patient died one month after admission because of respiratory insufficiency due to pleural effusion. Considering course and progression of disease, sCJD was suspected terminally. Analysis of the *PRNP* gene was not performed.

### Summary of clinical data

The age range of the patients was between 55 to 81 years at death and the duration of illness varied between 2 and 12 months (Table 1). The major clinical features were progressive dementia and gait disorder, ataxia, myoclonus, and extrapyramidal symptoms, including Parkinsonism in three, supported by decreased striatal DAT expression in two. Neither prominent sleep disorder nor polysomnography were reported or performed. EEG did not reveal periodic sharp wave complexes or permanent triphasic waves; CSF 14-3-3 was examined in three and showed positivity in all. Cranial MRI was performed in four; increased signal in the thalamus was observed in one. In five cases CJD was suspected clinically (in two only terminally) and in three could be classified as probable according to the WHO Surveillance criteria [27] prior to autopsy. Neither neurosurgery nor ophthalmic surgery had been performed; they had not received growth or gonadotropin hormone therapy. A positive family history for neuropsychiatric disorders was not reported in any of the cases.

**Table 1:** Overview of clinical data and performed examinations in the six patients

**Patient**	**Case 1**	**Case 2**	**Case 3**	**Case 4**	**Case 5**	**Case 6**
Age at death	77	55	81	74	62	80
Duration of illness	12	12	2	7	9	7
Sex	F	F	M	F	M	F
Codon 129	MM	MM	MM	MV	NA	NA
Symptoms						
Progressive dementia	+	+	+	+	+	+
Ataxia (Gait or Limb)	+	+	+	+	+	+
Parkinsonism	-	+	-	+	+	-
Myoclonus	+	+	+	+	-	-
Chorea	-	+	-	-	-	-
Pyramidal signs	+	+	-	-	-	-
Oculomotor disturbance	-	-	-	-	-	+
Cortical blindness	-	-	+	-	-	-
Clinical suspicion	CJD	CJD	CJD	CJD	DLB + ALC	WE/CJD
WHO Criteria (CJD)	Prob	Poss	Prob	Prob	*	-
Examinations						
CSF 14-3-3	+	NA	+	+	NA	NA
MRI	No	No	Yes	Yes	Yes	Yes
CDI (PM)	Yes	Yes	NA	Yes	NA	NA
WB (PM)	Yes	Yes	NA	Yes	NA	NA
Neuropathology						
BB stage	2	2	2	0	0	2
Thal Phase	No	1	1	No	No	1
Lewy body (B Stage)	No	No	No	No	No	No
phTDP-43	No	No	No	No	No	CA1 syn
Other	No	No	AG, MH	No	No	No

*Retrospectively could be evaluated as “possible CJD”. *Abbreviations:F* female, *M* male, *CJD* Creutzfeldt-Jakob disease, *DLB* dementia with Lewy bodies, *ALC* alcoholic encephalopathy, *WE* Wernicke encephalopathy, *AG* argyrophilic grains, *MH* microhaemorrhages in the hypothalamus, *NA* not available, *CA1-syn* synaptic deposits in hippocampus CA1. Thal phase indicates the phases (1–5) of the deposition of beta-amyloid in the brain [28]. BB indicates Braak and Braak stages of neurofibrillary degeneration [29].

### Neuropathological, immunohistochemical, and ultrastructural observations

Macroscopical evaluation of the brains revealed atrophy of the thalamus and additionally mild to moderate cerebral cortical atrophy in all lobes in case 1, 3 and 6, moderate atrophy in the caudate nucleus with mild depigmentation of the substantia nigra in cases 2 and 5, and a mild degree of atrophy in the frontal and temporal lobes in cases 4 and 5. There was a lack of macroscopic evidence of discoloration of the mammillary bodies or petechial haemorrhages in the medial thalamus or brainstem tegmentum.

#### Common histopathological features

**Figure 1:**
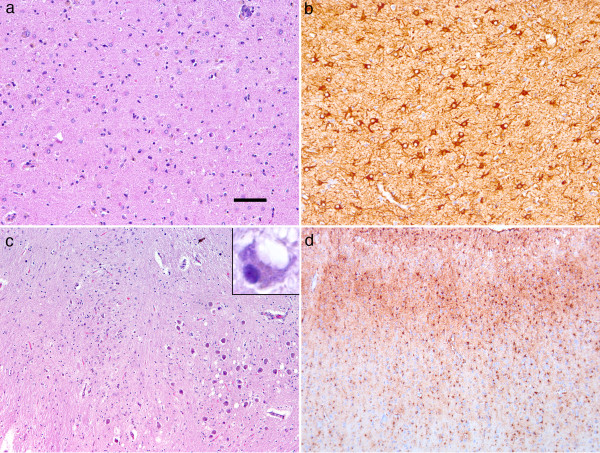
**Neuropathological observations.** Prominent neuronal loss and gliosis in the medial thalamus. (**a**: H&E, **b**: GFAP; representative image of case 1) associated with segmental gliosis and vacuolation in the inferior olives (**c**; representative image of case 5; inset demonstrates vacuolated neuron). Moderate gliosis mainly in the upper layers of the cortex (**d**; representative image of case 4). Bar in a indicates 50 μm for **a**, 30 μm for **b**, 100 μm for **c**, and 150 μm for **d**.

All six cases showed a relatively uniform histopathological phenotype (Table 2) characterised by prominent neuronal loss and reactive astrogliosis in the thalamus. In particular, the medial (Figure 1a,b), but also median and intralaminar nuclei were affected, while the anterior nucleus was only moderately affected, and lateral thalamic nuclei were not involved. In addition segmental gliosis of the inferior olives together with occasional vacuolated neurons (Figure 1c), and mild to moderate loss of neurons in the substantia nigra was noted. There was a lack or only subtle presence of classical spongiform change as described in CJD. However, a moderate degree of spongiosis of the superficial layers in the frontal, temporal, and parietal cortex as seen also in other neurodegenerative diseases (i.e. Alzheimer’s, dementia with Lewy bodies or frontotemporal lobar degeneration) was noted. Gliosis was moderate in the striatum, while neocortical areas were variably involved, in particular the second and third layers (Figure 1d).

**Table 2:** Summary of neuropathological observations

	**Case 1**			**Case 2**			**Case 3**			**Case 4**			**Case 5**			**Case 6**		
**Region**	**SC**	**G**	**PrP**	**SC**	**G**	**PrP**	**SC**	**G**	**PrP**	**SC**	**G**	**PrP**	**SC**	**G**	**PrP**	**SC**	**G**	**PrP**
Frontal Cx	+	++	++	+	+	+	-	-	+	-	+	+	-	++	-	+	++	+
Parietal Cx	-	+	-	+	+	++	-	+	+	-	+	++	-	++	++	+	++	+
Temporal Cx	-	+	+	-	+	-	-	-	-	-	+	+	-	-	-	-	-	-
Occipital Cx	-	+	+	-	++	+++	-	+	+++	-	+	+	-	++	++	NA	NA	NA
Entorhinal Cx	+	-	-	-	-	-	-	-	-	-	-	-	-	-	-	-	-	-
Hippocampus	-	-	-	-	-	-	-	-	-	-	-	-	-	-	-	-	-	-
Thalamus-Med	-	+++	-	-	+++	-	-	+++	-	-	+++	-	-	+++	-	-	+++	-
Thalamus-Lat	-	+	-	-	++	-	-	-	-	-	-	-	-	-	-	-	-	-
Thalamus-Ant	-	++	-	-	+	-	-	+	-	-	+	-	-	++	-	-	++	-
Caudate nucleus	-	++	-	-	++	-	-	+	-	-	++	-	-	++	+	-	+	-
Putamen	-	+	-	-	++	-	-	-	-	-	+	-	-	+	-	-	+	-
Globus pallidus	-	-	-	-	-	-	-	-	-	-	-	-	-	-	-	-	-	-
Substantia nigra	-	+	-	-	-	-	-	-	-	-	+	-	-	+	+	-	+	-
Dorsal raphe nucleus	-	+	-	-	+	-	-	+	-	-	++	-	-	+	-	-	-	-
Locus coeruleus	-	-	-	-	-	-	-	-	-	-	-	-	-	-	-	-	-	-
Pontine base nuclei	-	-	-	-	-	-	-	-	-	-	-	-	-	-	-	-	-	-
Inferior olive	-	++	-	-	++	-	-	+	-	-	++	-	+	+++	-	++	++	-
Dentate nucleus	-	-	-	-	-	-	-	-	-	-	-	-	-	-	-	-	+	-
Cbll Mol layer	-	-	-	-	-	+	-	-	+	-	-	-	-	-	-	-	-	-
Cbll Gran Layer	-	-	-	-	-	-	-	-	-	-	-	-	-	-	-	-	-	-
Purkinje cells	-	-	-	-	++	-	-	-	-	-	-	-	-	-	-	-	-	-

*SC* spongiform change; *G* gliosis; *PrP* immunoreactivity for PrP; *NA* not available; *Cx* cortex; *Med* medial; *Lat* lateral; *Ant* anterior; *Cbll* cerebellum. Semiquantitative scoring: – indicates none, +: mild, ++: moderate, +++: severe degree of changes or amount of immunoreactivity.

**Figure 2:**
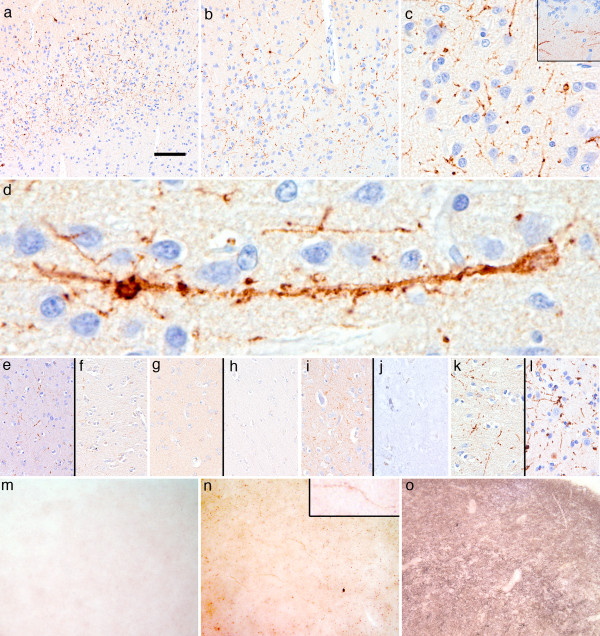
**Immunostaining for PrP.** Immunostaining for PrP revealed tiny granular deposits depicting neuronal processes in a linear or worm-like appearance predominantly in the 2nd and 3rd layers in neocortical regions. (**a-c**; representative images of case 2; right upper inset in c shows similar immunoreactivity in the cerebellum in case 3). Occasionally, small cell bodies were depicted as well **(d)**. These were detectable also by antibodies 6H4 **(e)**, and KG9 **(f)**, only focally by 3F4 **(g)**, and not by the anti-N-terminal BG4 antibody (**h** all representative images of case 4). The PrP immunoreactivity **(i)** was abolished after PK treatment (**j**; temporal cortex of case 1), while it was slightly more detectable when using only elongated citrate buffer pretreatment and omitting formic acid pretreatment (**k**: with, and **l**: without formic acid, represented by the occipital cortex of case 5). PET-blot examination performed using Thermolysin digested PrP^C^ completely from the frontal cortex brain tissue of a non-CJD control case **(m)**, while in the present cases the worm-like structures (enlarged in right upper inset) remained visible **(n)**, and in the cortex of a subject with CJD abundant PrP^Sc^ deposition was seen **(o)**. Bar in a represents 150 μm for **a**, 50 μm for **b**, **e-l**, 30 μm for **c**, 10 μm for **d**, and 400 μm for **m-o**.

**Figure 3:**
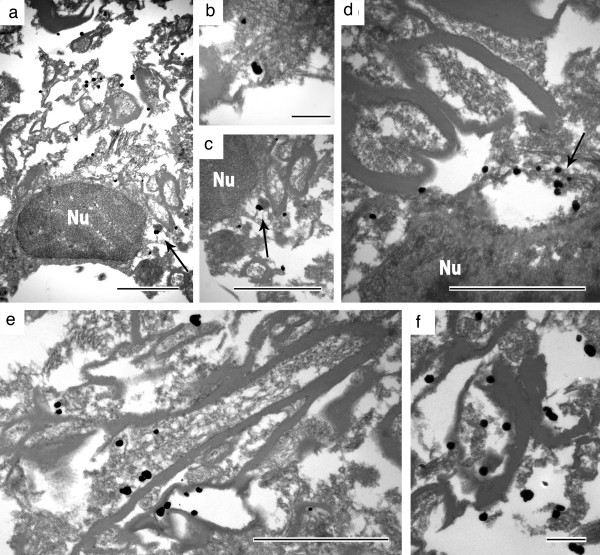
**Ultrastructural localisation of PrP using antibody 12F10 in the same frontal cortex section where light microscopical immunohistochemistry revealed linear and worm-like immunoreactivity.** Panel **a-d** demonstrates immunogold labeling specific for PrP mainly associated with endosome-like structures in the pericaryon (indicated by arrows; Nu: nucleus). Intraneuritic accumulation of PrP in longitudinal **(e)** and cross **(f)** sections of myelinated axons. Measuring bars represent 2 μm in **a**, **c**, **d**, and **e**, and 500 nm in **b** and **f**.

Immunostaining for PrP revealed a characteristic pattern in the neocortical regions. Tiny granular deposits apparently decorating branching neuronal processes in a linear or worm-like appearance with tiny varicosities were observed predominantly in the second and third layers (Figure 2a-c). In cases 2 and 3 we observed similar immunoreactivity in the molecular layer of the cerebellum parallel to the surface (Figure 2c inset). Occasionally, small cell bodies were depicted as well (Figure 2d). Plaque-like or intraneuronal inclusion-like deposits [21] were not seen. Neuritic deposits were best detected by 12F10, 6H4, L42, and KG9, only focally by 3F4, and not by the anti-N-terminal BG4 antibody (Figure 2e-h). Significantly, PK treatment completely abolished this staining (Figure 2i, j), while it was slightly more detectable when using only elongated citrate buffer pre-treatment and omitting formic acid pre-treatment (Figure 2k, l). Interestingly, PET-blot examination performed using Thermolysin digested PrP^C^ completely from the frontal cortex brain tissue of a non-CJD control case, while in the present cases the worm-like structures remained visible, and in the cortex of a subject with CJD abundant PrP^Sc^ deposition was seen (Figure 1m-o). PET-blot performed with PK was negative in a non-CJD control case and the present cases, but positive in a case of sFI and sCJD (Additional file 1: Figure S2). Ultrastructural examination of the same immunoreactivities in the same section (case 1) demonstrated intraneuritic localization of immuno-gold particles, partly associated with endosomal structures (Figure 3). A similar morphological appearance of PrP immunoreactivity was not observed in any of our definite CJD cases without selective thalamic degeneration, GSS, base pair insertion cases, or in the cases with other neuropathological diagnoses (see above). Accordingly, we defined this branching or linear neuritic immunoreactivity for senPrP^Sc^ as a specific feature for the six cases with thalamic degeneration. In none of the cases with Wernicke encephalopathy we observed similar PrP immunoreactivity. However, in two FFI cases with MM at codon 129 we observed similar deposits in the frontal and occipital cortex, while in the FFI case with MV at codon 129 this was very occasional and associated with synaptic deposits characteristic of PrDs (Additional file 1: Figure S3). Immunostaining for alpha-synuclein, phospho-TDP-43 and FUS did not reveal any pathological deposits, while anti-phospho-tau showed occasional tiny neuritic profiles in the neocortex. There was a lack of ischaemic/hypoxic damage in the cortical areas showing the peculiar PrP immunoreactivity; moreover, anti-APP did not demonstrate bulbs or axonal staining.

#### Additional alterations detected in some cases

Segmental loss of Purkinje cells was observed in case 2. A mild degree of neurofibrillary degeneration was noted in four cases (Braak & Braak stage II; Table 1). Scattered neurons with cytoplasmic p62 immunoreactive granular bodies were seen in the thalamus, and in cortical areas where PrP immunoreactivity was seen only in Patient 2, while other regions and brains of further subjects did not exhibit p62 inclusions. Case 6 (80 years-old) showed synaptic and thin neuritic phTDP-43 immunoreactive deposits in the CA1 subregion of the hippocampus. In case 3 tiny microhemorrhages in the hypothalamus/corpus mamillare reminiscent of Wernicke encephalopathy were observed, however, without obvious capillary proliferation. Periaqueductal regions of the brainstem did not show haemorrhages, and there was a lack of Prussian-blue positive blood pigments. The gliotic medial thalamus nuclei did not show haemorrhages or endothelial proliferation.

### Biochemical investigation

Frozen specimens of frontal cortex, occipital cortex and cerebellum from case 1, frontal cortex from case 2, and frontal cortex and cerebellum from case 4 were initially available for study and were used for the detection of PrP^res^ by Western blotting. A routine diagnostic Western blotting method involving digestion of a 10% w/v brain homogenate with 50 μg/ml proteinase K (PK), electrophoresis of 5–24 μl of the PK treated sample, and detection using the 3F4 anti-PrP antibody (epitope: residues 106–112 of the human PrP sequence) consistently failed to detect PrP^res^ in these samples (data not shown).

**Figure 4:**
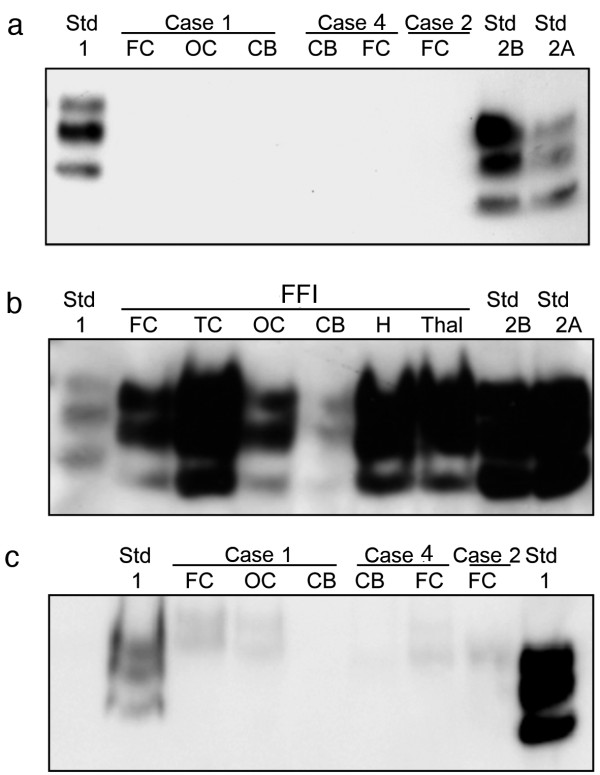
**Western blot analysis of present cases 1, 2, and 4 and an Austrian FFI case using 3F4 as the primary antibody.** PrP^res^ type 1, 2A and 2B standards were run in the lanes as indicated. **a**: PrP^res^ was undetectable in 200 μl (of 10% wt/vol brain homogenate) after centrifugal concentration. **b**: Centrifugal concentration (100 μl of 10% wt/vol brain homogenate) shows readily detectable PrP^res^ in the A-FFI case. **c**: PrP^res^ was undetectable in 500 μl of 10% wt/vol brain homogenate from cases 1, 2 and 4 after NaPTA precipitation. FC, frontal cortex; OC, occipital cortex; CB, cerebellum; TC, temporal cortex; H, hippocampus; Thal, thalamus.

Enrichment for PrP^res^ that might have been present at low abundance in these specimens was attempted using two different methods. First, centrifugation of PK treated brain homogenate, and second, sodium phosphotungstic acid (NaPTA) precipitation of abnormal prion protein and subsequent PK digestion, both enrichment methods followed by Western blot analysis using the 3F4 antibody. The centrifugal enrichment method used a starting volume of 200 μl but failed to detect PrP^res^ in the 18–30 kDa molecular mass region that is characteristic of CJD (Figure 4a). Similar analysis of 100 μl brain homogenate samples from a variety of regions from a case of FFI shows that the centrifugal enrichment method is sufficiently sensitive to detect PrP^res^ in this form of PrD, which is known to be characterised by relatively low levels of PrP^res^, compared to CJD (Figure 4b). NaPTA precipitation allows for the analysis of larger volumes of brain homogenate (500 μl). However, when frontal cortex, occipital cortex and cerebellum from case 1, frontal cortex from case 2, and frontal cortex and cerebellum from case 4 were analysed by this method, the only signal seen was a faint, poorly resolved band at around 30 kDa (Figure 4c). The possible significance of such faint bands was investigated by comparing one of these samples (frontal cortex from case 2) with similarly prepared and analysed samples from five non-CJD cases. The analysis failed to show any bands specific to the sample from case 2 (Additional file 1: Figure S4).

**Figure 5:**
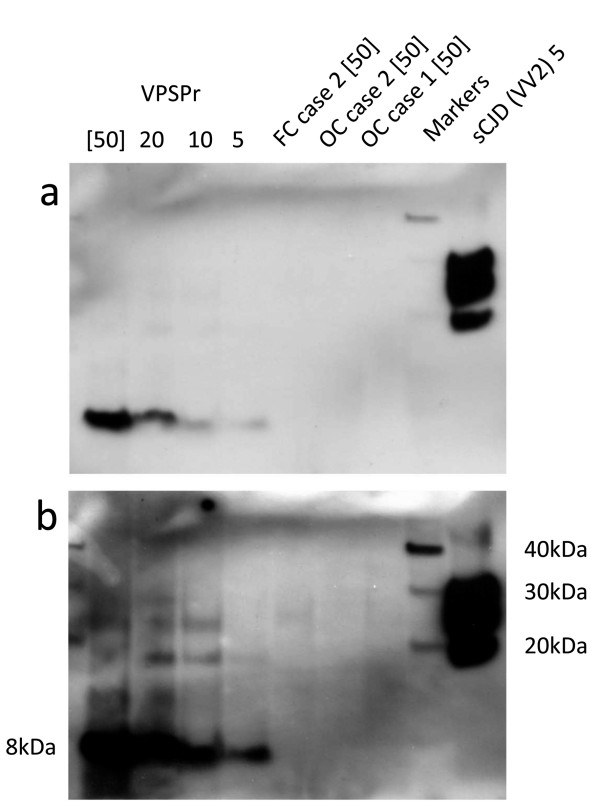
**Western blot analysis of PrP**^**res **^**in frontal cortex (FC) or occipital cortex (OC) of cases 1 and 2 compared to cortical samples of a variably protease sensitive prionopathy (VPSPr) case and a sporadic Creutzfeldt-Jakob disease of the VV2 subtype (sCJD (VV2))**. Sample loadings (μl of a 10% w/v brain homogenate) are shown for all lanes. Sample loading in square brackets ([]) denote volumes concentrated prior to loading. The positions molecular mass of marker proteins (Markers) and the low molecular mass PrP^res^ characteristic of VPSPr are given in kilodaltons (kDa). **(a)** and **(b)** show short (3 minutes) and long (30 minutes) exposures of the same Western blot.

The recently described prion disease VPSPr is characterised by poorly protease resistant prion protein that is detected in the form of a low abundance 8 kDa PrP^res^ fragment accompanied by a faint ladder of bands extending into the 18–30 kDa region. Comparison of samples of occipital cortex from case 1 and frontal and occipital cortex from case 2 with a frontal cortex sample from a known case of VPSPr using centrifugal enrichment from 50 μl of a 10% w/v brain homogenate failed to provide any evidence of an 8 kDa band characteristic of VPSPr in case 1 or case 2 (Figure 5a). Prolonged exposure of the same blot, which included direct loading of 20, 10 and 5 μl of the 10% w/v VPSPr brain homogenate, indicated that if the 8 kDa band is present it must be at a level ten times less than in the VPSPr sample (Figure 5b). Prolonged exposure of these blots did however result in the presence of some very faint bands in the 8–30 kDa region. The possible significance of such faint bands was investigated by comparing one of the samples (frontal cortex from case 2) with similarly prepared and analysed samples from five non-CJD cases. The analysis failed to show any bands specific to the sample from case 2 (Additional file 1: Figure S5).

**Figure 6:**
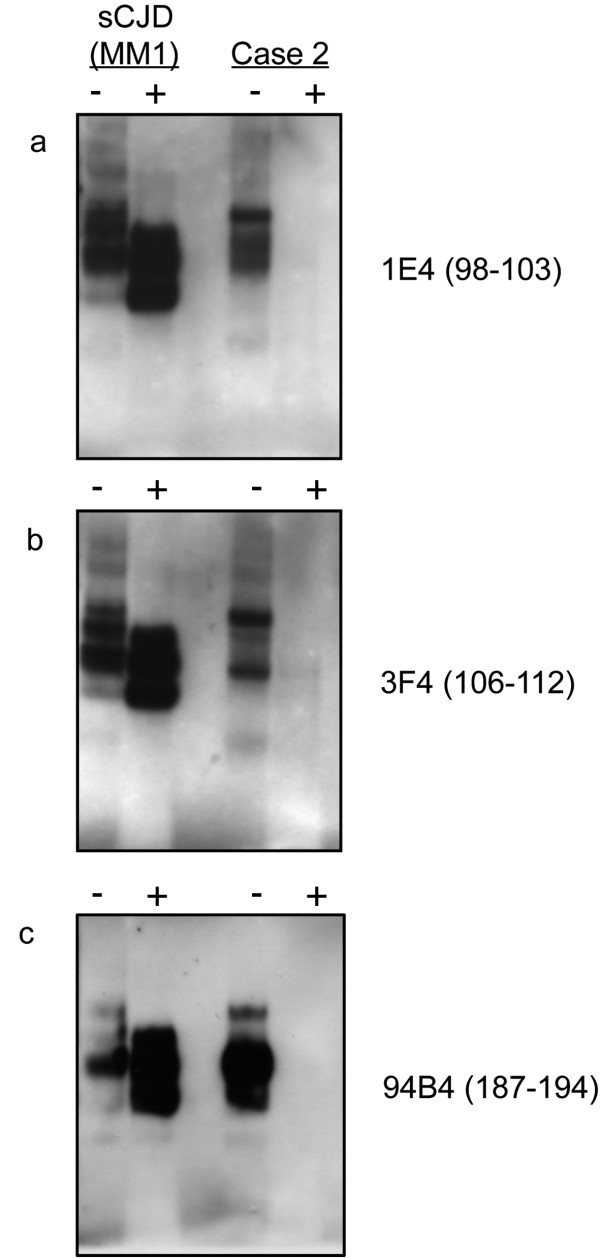
**Western blot analysis of grey matter enriched frontal cortex from a sCJD MM1 patient and case 2 using anti-PrP antibodies 1E4. (a)**, 3F4 **(b)** and 94B4 **(c)** (epitopes as indicated on the figure). Non-PK treated samples (−) were loaded at 6 μl/lane of 10% w/v brain homogenate, whereas PK-treated samples (+) were loaded at the maximum of 24 μl/lane of 10% w/v brain homogenate, but without any sample concentration pre-treatment.

The 1E4 anti-PrP antibody (epitope residues 98–103 of the human PrP sequence) has been reported to have a high sensitivity for PrP^res^ detection in Western blotting of VPSPr brain. However, the use of this antibody failed to detect any additional bands in Western blot analysis of 24 μl of 10% w/v frontal cortex homogenate from case 2 (Figure 6a) as compared with use of the 3F4 antibody on a duplicate blot (Figure 6b). The possibility of extensive N-terminal truncation of PrP^res^ and consequent loss of the 3F4 and 1E4 epitopes was tested by screening a further replicate Western blot with the monoclonal antibody 94B4 (epitope residues 187–194 of the human PrP sequence). However, this approach failed to provide evidence of C-terminal PrP^res^ fragments in the frontal cortex homogenate from case 2 (Figure 6c).

Conformation dependent immunoassay (CDI) using a capture antibody to a discontinuous C-terminal epitope (MAR-1) and detection using a Europium conjugated detection antibody (3F4) that recognises its epitope in denatured, but not native PrP^Sc^ was then used as an alternative sensitive PrP^Sc^ detection method that does not necessarily depend on the presence of protease-resistant abnormal PrP. The time resolved fluorescence read-out of the CDI assay was calibrated using recombinant human prion PrP and the results expressed as μg PrP/g of brain sample analysed. The values obtained under native conditions were taken to be a measure of normal cellular prion protein (PrP^C^), whereas any increase in values obtained following denaturation with guanidine hydrochloride (D-N) were taken to indicate the presence of PrP^Sc^.

**Figure 7:**
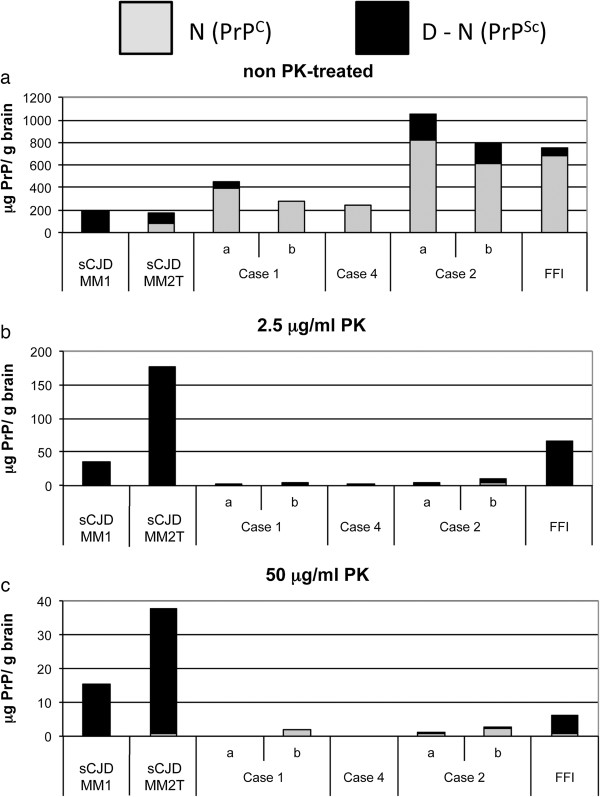
**CDI analysis of the frontal cerebral cortices of the case 1, 2 and 4, and an FFI case after treatment with 0, 2.5 and 50 μg/ml PK.** (panels **a**, **b** and **c**, respectively). Frontal cortical brain homogenates from cases of sporadic Creutzfeldt-Jakob disease (sCJD) types MM1 and MM2T (the latter also known as sporadic fatal insomnia, sFI) were also analysed for comparison. Note that the range of the y-axis decreases from panel a to b to c. For case 1 and 2 duplicate frozen samples, labeled ‘a’ and ‘b’, were available for CDI analysis.

CDI analysis of frontal cortex specimens from cases 1, 2 and 4 all showed high native (N) CDI values indicative of abundant PrP^C^ and in one sample from case 1 and two samples from case 2 positive denatured minus native values (D-N) were seen, indicative of the presence of PrP^Sc^ (Figure 7a). Positive D-N values were seen in frontal cortex samples from sCJD cases of the MM1 and “thalamic variant” (MM2T) or sFI subtypes and from a case of FFI (Figure 7a). Treatment of brain samples with a low level of PK (2.5 μg/ml) prior to CDI analysis effectively abolished the N values in all samples indicating the removal of PrP^C^. D-N values for the samples from cases 1, 2, and 4 were markedly reduced by mild PK treatment, whereas a considerable D-N value remained after treatment of sCJD and FFI samples with PK at this level (Figure 7b). A proportion of this D-N signal in the cases of CJD and FFI resisted digestion with higher levels of PK (50 μg/ml), whereas the D-N values from the samples of cases 1, 2 and 4 became very low indeed (Figure 7c).

It remained possible that brain regions other than frontal cortex might contain more readily CDI-detectable PrP^Sc^ in cases 1, 2 and 4. Samples of additional brain regions were obtained for case 2 and these were analysed by CDI without prior PK treatment or with mild PK treatment (2.5 μg/ml). In no brain region of case 2 (including the thalamus) did the D-N value exceed that found in the frontal cortex when CDI was performed in the absence of PK treatment (Additional file 1: Figure S6a). Following low-level PK treatment, the D-N values of all regional samples were again markedly reduced (Additional file 1: Figure S6b).

**Figure 8:**
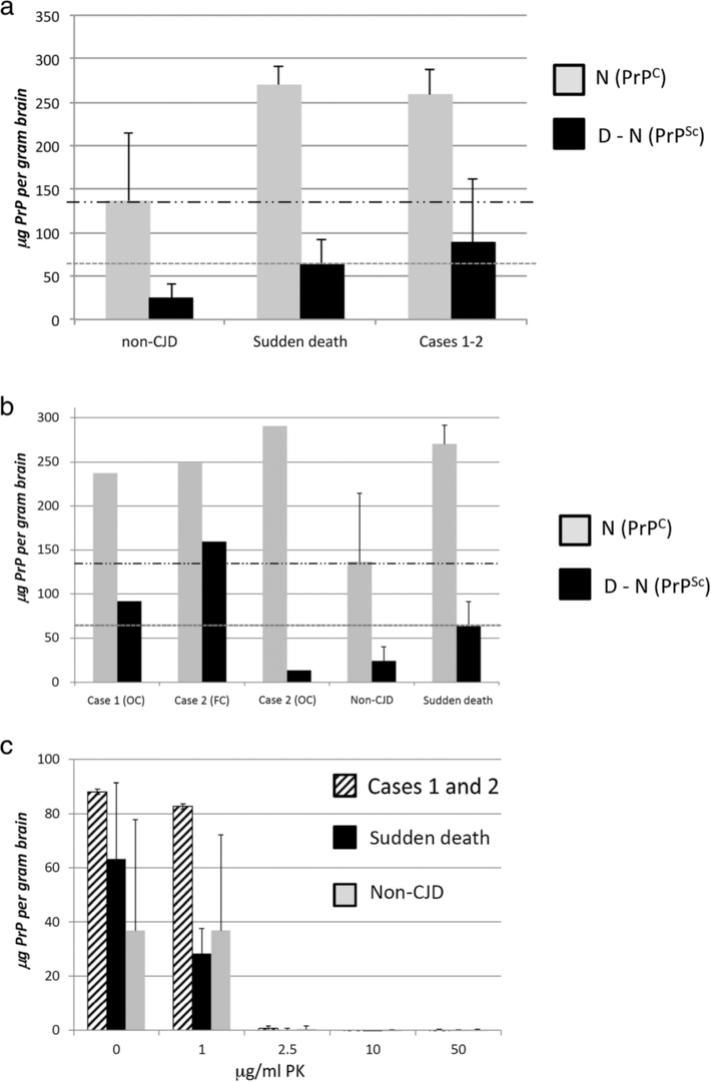
**CDI analysis of cases compared with negative control groups. (a)** Comparison of CDI values (means) for non-CJD control, sudden death control and cerebral cortex samples. Frontal cortex samples from ten cases from the Medical Research Council Edinburgh Brain and Tissue bank were analysed by CDI as controls. Five of these were Sudden Death cases with no history of a neurological condition (‘Sudden death’), whereas another five were cases initially referred to NCJDRSU as suspected CJD cases, but given an alternative final diagnosis (‘non-CJD’). These samples were analysed in comparison with three samples of cerebral cortex from cases 1 (one sample) and 2 (two samples). The upper and lower dashed lines indicate the D-N cut-off values, these being the [mean + (2.5 X Standard Deviation)] for sudden death and non-CJD cerebral cortex tissue, respectively. **(b)** Comparison of CDI values (means) for non-CJD control and sudden death control versus the individual samples from cases 1 and 2. **(c)** The D-N values are shown from CDI analysis of a sample of frontal cortex from cases 1 and 2 (striped bar) and sudden death cases (black bar) or non-CJD neurological control cases (grey bars), following treatment with various concentrations of PK as indicated on the x-axis.

In order to establish whether the low D-N values determined for frontal cortex of case 2 (Figure 7a) were meaningful and represented the detection of PrP^Sc^, we conducted CDI analysis using frontal cortex samples from two carefully selected negative control groups. Five non-CJD cases and five cases of sudden death were analysed and used to establish cut off values (mean plus 2.5 times the standard deviation). Whether examined as a group (Figure 8a) or considered as individual samples (Figure 8b) the D-N values of frontal cortex samples from cases 1 and 2 were at best only borderline positive with respect to these cut-off values. The introduction of low level PK treatment prior to CDI analysis fails to provide any better distinction between the D-N values from frontal cortex from case 2 and the two negative control groups. In each group the D-N values fell abruptly between treatment with 1 μg/ml and 2.5 μg/ml PK (Figure 8c).

In summary, Western blotting using a range of relevant anti-PrP antibodies (to detect different PrP^res^ fragments) and two different sample enrichment methods (to increase PrP^res^ detection sensitivity) failed to produce evidence of PrP^res^ in these cases, similar to that which is found in CJD, sFI, FFI or VPSPr (Figures 4, 5 and 6). CDI analysis using a range of PK digestion conditions (and no PK digestion) produced only very limited evidence of meaningful D-N values, indicative of the presence of PrP^Sc^ in these cases, when the results were compared with appropriate negative control groups (Figures 7 and 8).

## Discussion

### General context

Distinct neurodegenerative diseases are characterised by progressive loss of neurons predominantly from specific anatomical regions, and this presumably accounts for the specific focal deficits and clinical features. A further feature is the deposition of a limited range of proteins, and the particular proteins involved are the basis of the molecular pathological classification [30]. This latter concept has implications for biomarker development, be it either using body fluids or substances that allow *in vivo* detection of pathological protein deposition in the brain (i.e. Pittsburgh compound B is already used for visualizing amyloid-β in Alzheimer’s disease) [31]. However, there are numerous disease-associated protein modifications, but their pathogenetic relevance is not yet understood fully. The presence of PK-resistant PrP was for many years considered the defining molecular feature of PrDs, until CDI showed that disease-associated PrP (PrP^Sc^) actually exists in PK-sensitive forms in addition to the more familiar PK-resistant forms and that PK-sensitive forms of PrP^Sc^ usually predominate [7,32]. The significance of senPrP^Sc^ was supported and extended by the description of the human disease VPSPr, in which PrP^Sc^ is readily detectable, but PrP^res^ is found only at low and variable levels with a highly characteristic “ladder-like” appearance in immunoblotting [10,11].

On the basis of their clinico-pathological phenotype we believe that the six cases reported here (1) are sufficiently similar to be considered as representing examples of the same disease entity, (2) have clinical and neuropathological in common with human prion disease, (3) fall outside the currently known spectrum of human prion diseases, but (4) lack a plausible alternative diagnosis. It should be noted that in cases 5 and 6 genetic analysis could not be performed, thus, in spite the lack of family history for a similar disorder, we cannot exclude the possibility that these cases represent atypical forms of FFI. Furthermore, in these cases and case 3 we could not evaluate biochemical alterations in detail and thus we are forced to base our conclusions primarily on the neuropathological evaluation of this group of cases as a whole.

### Biochemical aspects

One of the consistent features of the cases described here is the presence of a highly unusual immunostaining pattern for PrP in the cerebral cortex. The key question that follows from this observation is, whether this novel immunostaining pattern is accompanied by the presence of PrP in these cases in a form that is recognised to occur in known PrDs. Intensive investigation using the best available methods seems to suggest that it does not. If disease associated PrP is present in these cases it must be i) in samples or brain regions that were not analysed biochemically, ii) at levels below the detection limits of Western blotting and CDI, or iii) in a form that is indistinguishable from PrP^C^ in Western blotting and CDI as employed here. There is a growing recognition that the analysis of highly protease-resistant PrP in the brain in PrD does not provide a full description of the abnormal forms of PrP present in the tissue and that protease sensitive forms of PrP play a role in pathogenesis and transmissibility [33]. The situation that we report here could potentially represent a very extreme example of this phenomenon. If true it seems to indicate that end stage disease (i.e. in the present cases with thalamic degeneration) can be reached without gross accumulation of highly protease-resistant PrP. Whether prionopathies that lack detectable PrP^res^ in the brain are transmissible (a major feature of PrDs), needs to be determined, and transmission studies have been initiated involving one of our cases.

### Clinical features

The rapidly progressive clinical course raised the suspicion of PrD in three out of the six subjects. Indeed, according to WHO Surveillance criteria [27] these cases were classified as probable CJD, including detection of protein 14-3-3 in the CSF. This is different from the thalamic form of sCJD (i.e. sFI) [9]. Furthermore, there was no evidence of a sleep disorder or autonomic dysfunction either, which would have raised this diagnosis or would have initiated a polysomnographic examination during life. Parkinsonism, which is also a feature of the VPSPr cases [10], was a notable finding in some cases, and was supported by SPECT examination of the basal ganglia in two patients. These clinical observations were compatible with the loss of neurons in the substantia nigra and gliosis in the striatum, although the degree did not reach that seen in Parkinson’s disease or other neurodegenerative disease with Parkinsonism. One case was classified as dementia with Lewy bodies on clinical criteria and only the neuropathological evaluation clarified the diagnosis. This emphasises the need for systematic neuropathological evaluation in the clinical setting of rapidly progressive Parkinsonism with dementia, even when supported by imaging findings, since dementia with Lewy bodies may have a rapidly progressive form also [34]. In one subject (case 2) the possibility of Huntington’s disease was also raised, and in another patient (case 4) hydrocephalus was observed. In the latter, clinical examinations did not show increased pressure and no clinical improvement was observed after taking 30 ml CSF, but rather there was a decreased DAT expression in the striatum. There is a spectrum of disorders, which may mimic PrDs [35-37] and which were excluded both during clinical examinations and neuropathology. In sum, rapid dementia is a common feature of our cases, which show some variability in other symptoms; concomitant pathologies, which are frequent in the elderly, may influence the symptoms [38].

### Epidemiology

We note that in the period between 1993 and 2013, a total of 249 definite CJD cases were diagnosed (sporadic, iatrogenic, genetic) in Austria as part of PrD Surveillance. However, we did not observe the thalamic type of CJD (sFI) in this cohort. The fact that our six cases with a similar neuropathological phenotype were diagnosed within two years is striking but should be interpreted with caution; in particular the frequency of rare diseases may vary considerably when analysed on a yearly basis. Follow-up studies are required to clarify these epidemiological aspects. Interestingly, in contrast to sFI cases, which are associated with MM at codon 129, one of the present cases was MV. On a neuropathological level, we could not see any major differences between this MV case and the other cases.

### Pathological diagnosis

Immunostaining for disease-associated PrP has become a standard for the definitive diagnosis of PrDs. There are different patterns, including synaptic, patchy/perivacuolar, perineuronal, or plaque types, which can be detected by an enhanced pretreatment method that includes formic acid or PK and autoclaving [17]. Indeed, these patterns correlate well with recognised molecular subtypes [39]. Disease-associated PrP, detectable also after PK-treatment, may also deposit intraneuronally usually associated with the endosomal-lysosomal system but also rarely forming inclusion bodies [21,40]. In addition, a recent study suggested that filamentous PrP immunoreactivity may be a common feature of genetic PrDs [41]. In the present cases we observed none of the aforementioned immunostaining patterns, but instead we observed a neuritic distribution as a new pattern, which appeared uniformly in each of our six cases and was not detected in our surveillance cohort of various human PrDs or in a cohort of non-PrD cases, which were processed and evaluated with the same methods. Interestingly, only in the examined FFI cases we detected similar cortical PrP deposits in addition to classical synaptic type immunoreactivity. This could support a relationship to FFI. Alternatively, it could be theorised that damage to the thalamic neurons causes secondary damage to thalamocortical projections. Although in 8 cases with thalamic pathology we did not observe similar immunoreactivities, we cannot exclude this possibility. Still this indicates that PrP may undergo conformational change, which expands beyond the well-established morphologies. In a single case we detected microhaemorrhages in the hypothalamus suggestive of Wernicke-encephalopathy. However, we did not observe signs of a chronic form of Wernicke encephalopathy, moreover in six cases with classical morphological features of Wernicke encephalopathy, there was a lack of similar PrP immunoreactivity in the cortex. Interestingly, Wernicke encephalopathy may associate with sCJD [42]. Although reminiscent of the recently reported filamentous PrP immunoreactivity [41], the peculiar immunoreactivity in our cases was confined to the 2nd and 3rd layers of the neocortex or occasionally to the molecular layer of the cerebellum but was never observed in the white matter. The peculiar branching type of cortical immunoreactivity in our series of cases was abolished by PK-treatment of sections and somewhat increased by the omission of the formic acid pre-treatment. Interestingly, some samples in the above study on filamentous PrP immunoreactivity [41] also lacked PK-resistant PrP; however it was not stated whether samples showing the immunoreactivity were evaluated or not [41]. The neuritic immunoreactivity in our cases was not uniformly detectable by all of the anti-PrP antibodies used. Thus, care must be taken which anti-PrP antibody is used when evaluating cases with thalamic degeneration without alternative pathologies. In our ultrastructural analysis, we were able to examine the same section used for light microscopy and electron microscopy [21], and demonstrated the presence of PrP immunoreactivity associated with endosomes, which are crucial players in the processing of PrP [43]. The lack of detection using the N-terminal anti-PrP antibody also suggests that PrP undergoes processing (i.e. truncation) within the cell. Furthermore, the unique detection of this type of PrP immunoreactivity in PET-blot after thermolysin, but not following PK treatment, supports the notion that this neuritic PrP immunoreactivity pattern could be the immunomorphological representative of a disease-associated PK-sensitive PrP conformer (senPrP^Sc^). Thermolysin has previously been shown to digest PrP^C^ leaving both PK sensitive and resistant PrP^Sc^ isoforms undigested [20]. The concept that some PrP deposits may contain PK-sensitive PrP is further supported by recent observations in a genetic prion disease case carrying a 144 base pair insertion [44]. There are further immunostaining patterns associated with non-PrDs that are thought to represent PrP^C^[45,46]. A similar neuritic pattern has not been described; moreover, in our cases we excluded ischaemic/hypoxic damage as a potential cause for PrP^C^ upregulation. Additionally, the lack of neuritic anti-APP immunoreactivity was not suggestive of a defect of the acute axonal transport. Thus, in addition to the already characterised immunomorphology of physiological cellular PrP [45] and disease-associated PrP with corresponding PK-resistance in immunoblottting [17], further comparative studies are needed to characterise the spectrum of immunoreactivities that represent conformationally altered (“disease-associated”) PrP forms with PK-sensitivity (i.e. senPrP^Sc^). In 2005, Safar *et al*. suggested that CDI is substantially more sensitive than immunohistochemistry [7]. This report places some constraints on the interpretation of CDI in a diagnostic role and shows how immunohistochemistry remains useful especially in atypical cases such as these.

## Conclusion

In conclusion, we present here six cases with a uniform neuropathological phenotype (thalamic degeneration with cortical neuritic PrP immunoreactivity) associated with a fatal clinical disease and rapidly progressive dementia and (where tested) with apparently PK-sensitive disease-associated PrP. The neuropathological phenotype shows similarities with sporadic and familial fatal insomnia. In the present study we cannot conclude whether the observed biochemical phenotype is related to the spectrum of atypical prionopathies or reflects a completely different disease-process. Whether the present cases represent a PK-sensitive subtype of sporadic fatal insomnia (also called MM type 2-thalamic) or a different entity, possibly even unrelated to the PrDs, merits further study. Clinico-pathological aspects of these cases have implications for the differential diagnosis and pathogenesis of neurodegenerative diseases, as well as for surveillance of human prion diseases. Neurodegeneration may be present in the brain without prominent pathological protein depositions.

## Competing interests

The authors report no competing interests.

## Author’s contributions

GGK, SW, ASB, RH, JWI and HB carried out the histological and PET-blot analysis and collated the neuropathological data. AP, HY, AG, MWH carried out the protein and CDI analysis and Western blotting and collated the biochemical data. SK, RK, DL, HA, EZ, EH, AG, AM, and MK recruited, clinically and neuroradiologically examined the patients. TS carried out the genetic analysis. TV performed laboratory diagnostic investigations. LL and GP performed the ultrastructural examination. GGK, AP, and MWH wrote the manuscript. GGK, AP, JWI, LL, MWH, and HB critically read and revised the manuscript. All authors read and approved the final manuscript.

## Supplementary Material

Additional file 1: Figure S1 a. Cranial MRI image (Case 3). b. Cranial MRI images (Case 4). c. EEG (Case 4). d. Cranial MRI (case 6). e. DWI images (Case 6). **Figure S2.** PET blot examination following PK digestion of a section from the entorhinal cortex in a case of sFI, and case 2 from the present series. **Figure S3.** Immunostaining for PrP (antibody12F10) in three cases of FFI reveals focally similar neuritic immunoreactivity. **Figure S4.** Western blot analysis of PrPres in frontal cortex of the thalamic case 2 compared to cortical samples of known non-CJD neurological control cases, a variably protease sensitive prionopathy (VPSPr) case and a sporadic Creutzfeldt-Jakob disease of the VV2 subtype. Sample loadings (μl of a 10% w/v brain homogenate) are shown for all lanes. Sample loading in square brackets ([]) denote volumes used for NaPTA precipitation prior to PK digestion. **Figure S5.** Western blot analysis of PrPres in frontal cortex (FC) of the case 2 compared to cortical samples of known non-CJD neurological control cases, a variably protease sensitive prionopathy (VPSPr) case and a sporadic Creutzfeldt-Jakob disease of the VV2 subtype (sCJD (VV2)). Sample loadings (μl of a 10% w/v brain homogenate) are shown for all lanes. Sample loading in square brackets ([]) denote volumes concentrated prior to loading. The positions of the molecular mass of marker proteins (Markers) and the low molecular mass PrPres characteristic of VPSPr are given in kilodaltons (kDa). (a) and (b) show short (3 minutes) and long (30 minutes) exposures of the same Western blot. **Figure S6.** CDI analysis of brain regions of case 2 after treatment with 0 and 2.5 μg/ml PK. Click here for file
