# Supplementary material for: Rapidly progressive dementia with thalamic degeneration and peculiar cortical prion protein immunoreactivity, but absence of proteinase K resistant PrP: a new disease entity?
Source: Acta Neuropathol Commun. 2013 Nov 11;1:72. doi: 10.1186/2051-5960-1-72 (PMC3835463; doi:10.1186/2051-5960-1-72)

LEGENDS TO SUPPLEMENTARY FIGURES

Supplementary Figure 1.

a. Cranial MRI image (Case 3).

b. Cranial MRI images (Case 4). FLAIR (left) and T2 (right) sequence 3 months before death: note the ventricle enlargement.

c. EEG: general slowing of activity with triphasic waves without periodicity.

d. The coronal FLAIR sequence displays bilateral thalamic hyperintensities (case 6).

e. DWI images showing signal alterations (arrows) in both thalami (left) and the frontoparietal cortex (right) (Case 6).

Supplementary Figure 2. PET blot examination following PK digestion of a section from the entorhinal cortex in a case of (a) sFI, showing focal positive labelling for protease-resistant PrP (dark) and (b) case 2 from the present series, showing no labelling. PrP labelling was performed using the 12F10 anti-prion protein antibody.

Supplementary Figure 3.

Immunostaining for PrP (antibody12F10) in three cases of FFI reveals focally similar neuritic immunoreactivity.

Supplementary Figure 4.

Western blot analysis of PrP^{res} in frontal cortex (FC) of the thalamic case 2 compared to cortical samples of known non-CJD neurological control cases, a variably protease sensitive prionopathy (VPSPr) case and a sporadic Creutzfeldt-Jakob disease of the VV2 subtype (sCJD (VV2)). Sample loadings (µl of a 10% w/v brain homogenate) are shown for all lanes. Sample loading in square brackets ([]) denote volumes used for NaPTA precipitation prior to

PK digestion. The positions of molecular mass of marker proteins (Markers) are given in kilodaltons (kDa).

Supplementary Figure 5.

Western blot analysis of PrP^{res} in frontal cortex (FC) of the case 2 compared to cortical samples of known non-CJD neurological control cases, a variably protease sensitive prionopathy (VPSPr) case and a sporadic Creutzfeldt-Jakob disease of the VV2 subtype (sCJD (VV2)). Sample loadings (µl of a 10% w/v brain homogenate) are shown for all lanes. Sample loading in square brackets ([]) denote volumes concentrated prior to loading. The positions of the molecular mass of marker proteins (Markers) and the low molecular mass PrP^{res} characteristic of VPSPr are given in kilodaltons (kDa). (a) and (b) show short (3 minutes) and long (30 minutes) exposures of the same Western blot.

Supplementary Figure 6. CDI analysis of brain regions of case 2 after treatment with 0 and 2.5µg/ml PK (panels **a** and **b**, respectively). Duplicate frozen samples, labeled ‘a’ and ‘b’, were available for CDI analysis. OC, occipital cortex; CB, cerebellum; PC, parietal cortex; Thal, thalamus; BG, basal ganglia; P, pons; Me, medulla oblongata.

Supplemental Figure 1.

a. Cranial MRI images (FLAIR, Case 3).

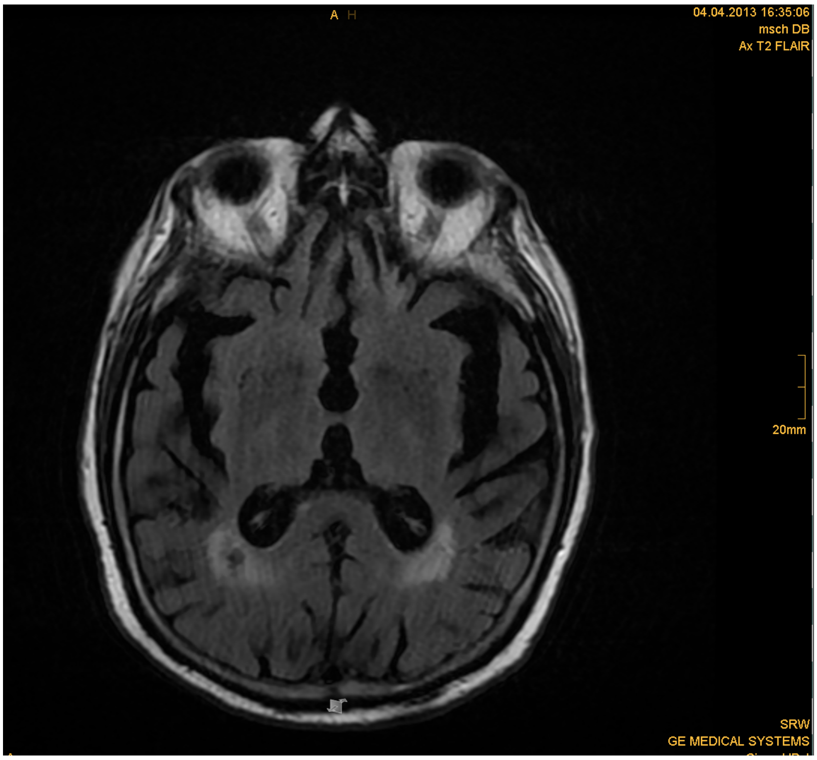

b. Cranial MRI images (Case 4). FLAIR (left) and T2 (right) sequence 3 months before death: note the ventricle enlargement.

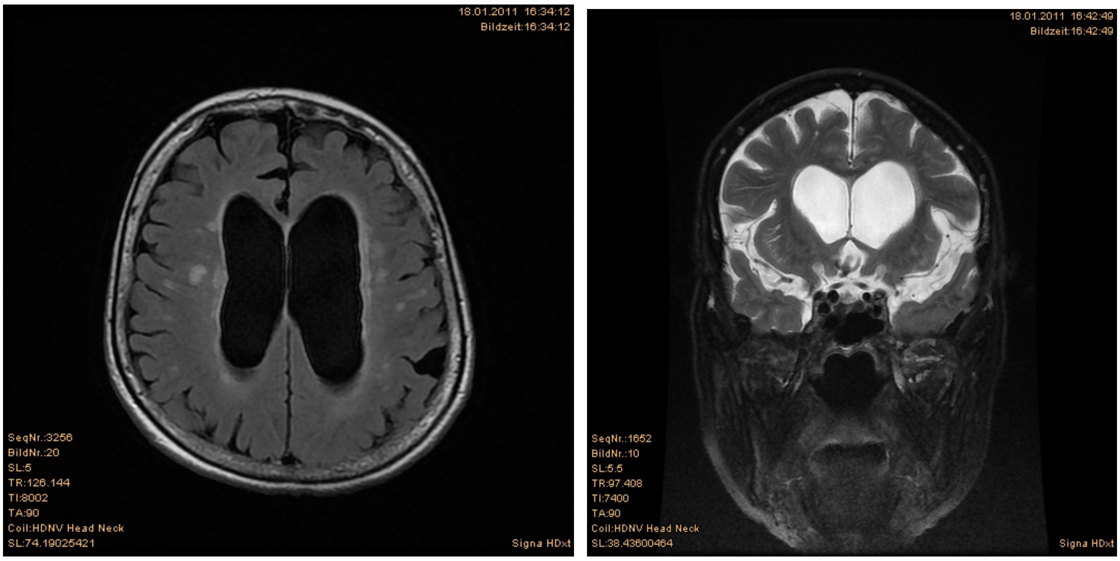

c. EEG: general slowing of activity with triphasic waves without periodicity (Case 4).

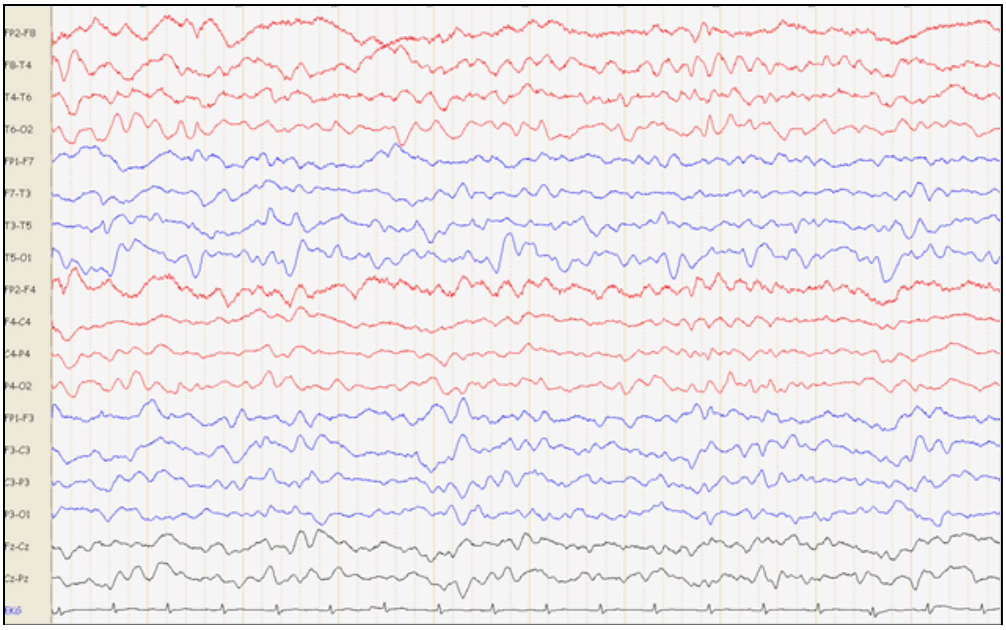

d. The coronal FLAIR sequence displays bilateral thalamic hyperintensities (case 6).

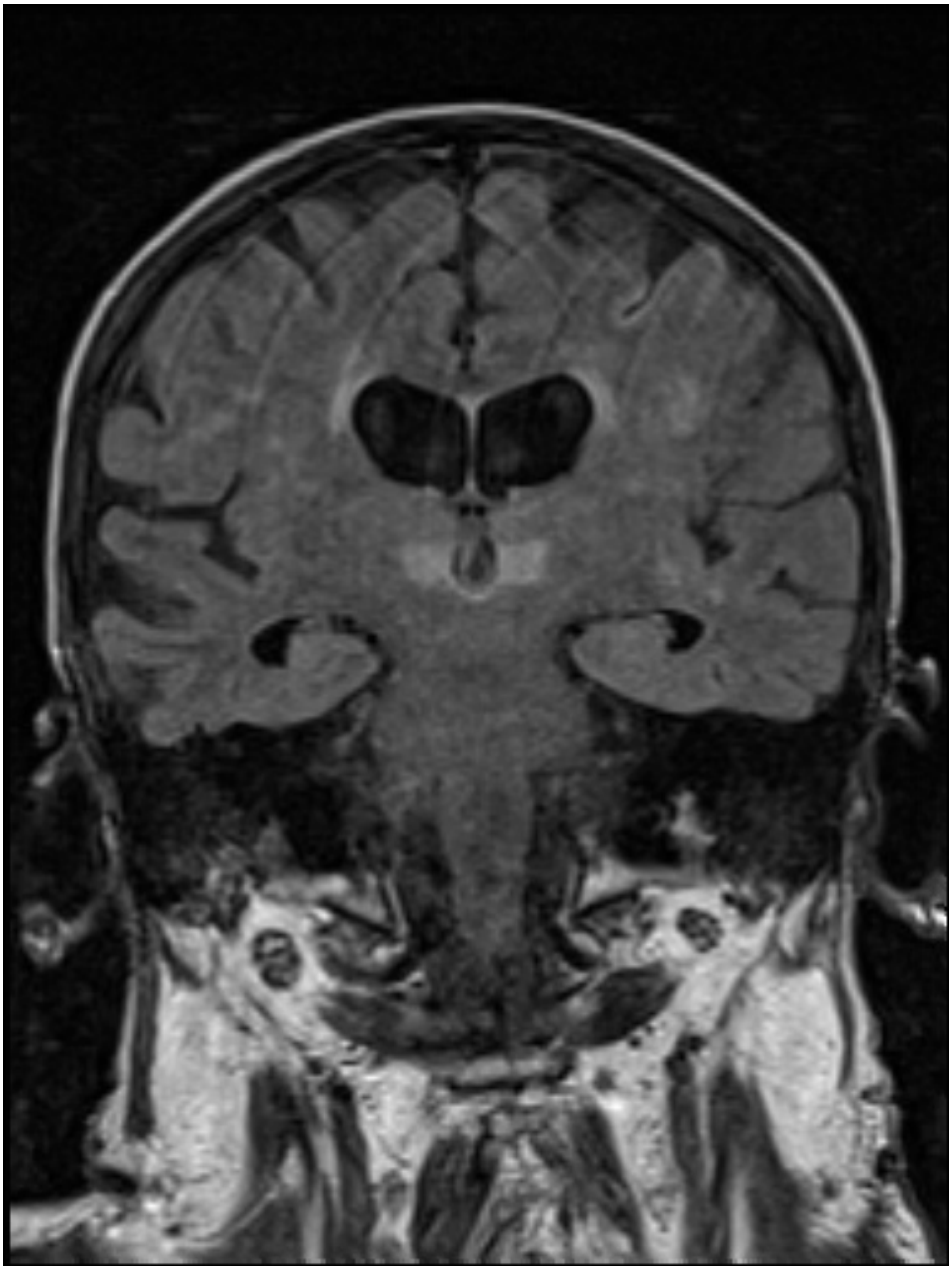

e. DWI images showing signal alterations (arrows) in both thalami (left) and the frontoparietal cortex (right) (Case 6).

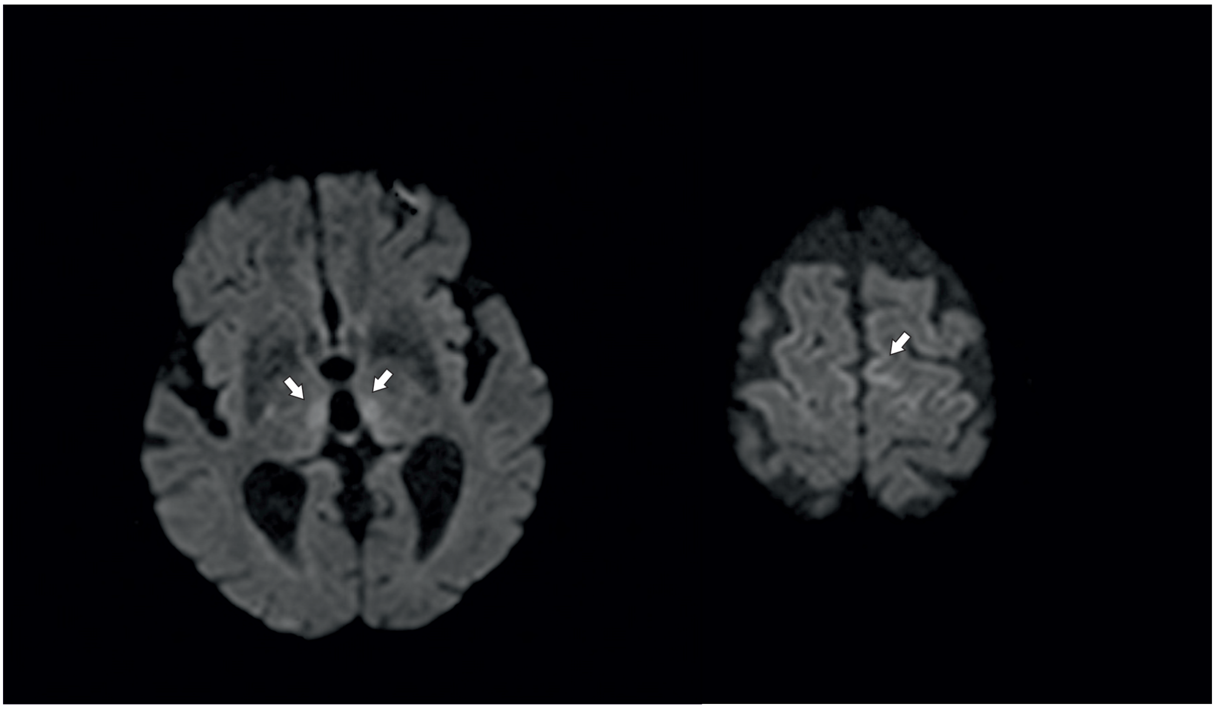

Supplemental Figure 2

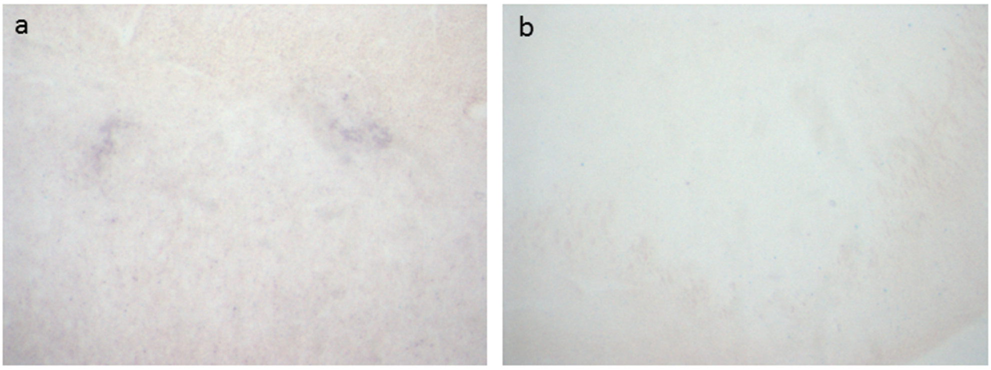

PETblot examination following PK digestion of a section from the entorhinal cortex in a case of (a) sFI, showing focal positive labelling for protease-resistant PrP (dark) and (b) case 2 from the present series, showing no labelling.
PrP labelling was performed using the 12F10 anti-prion protein antibody.

Supplemental Figure-3

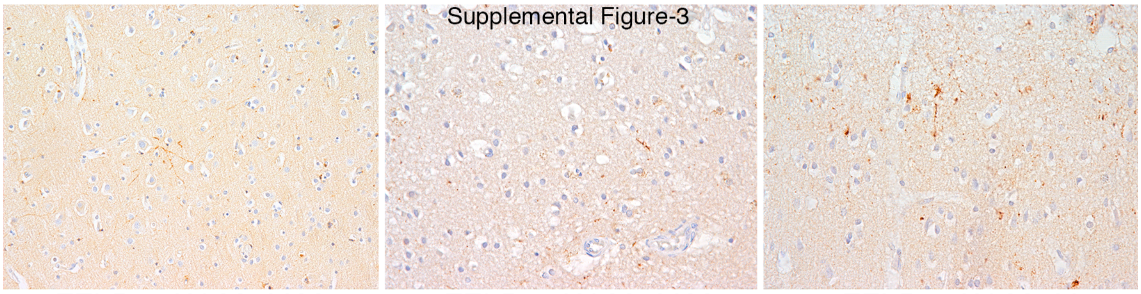

FFI-D178N-M129M　　FFI-D178N-M129M　　FFI-D178N-M129v

Supplementary figure 4

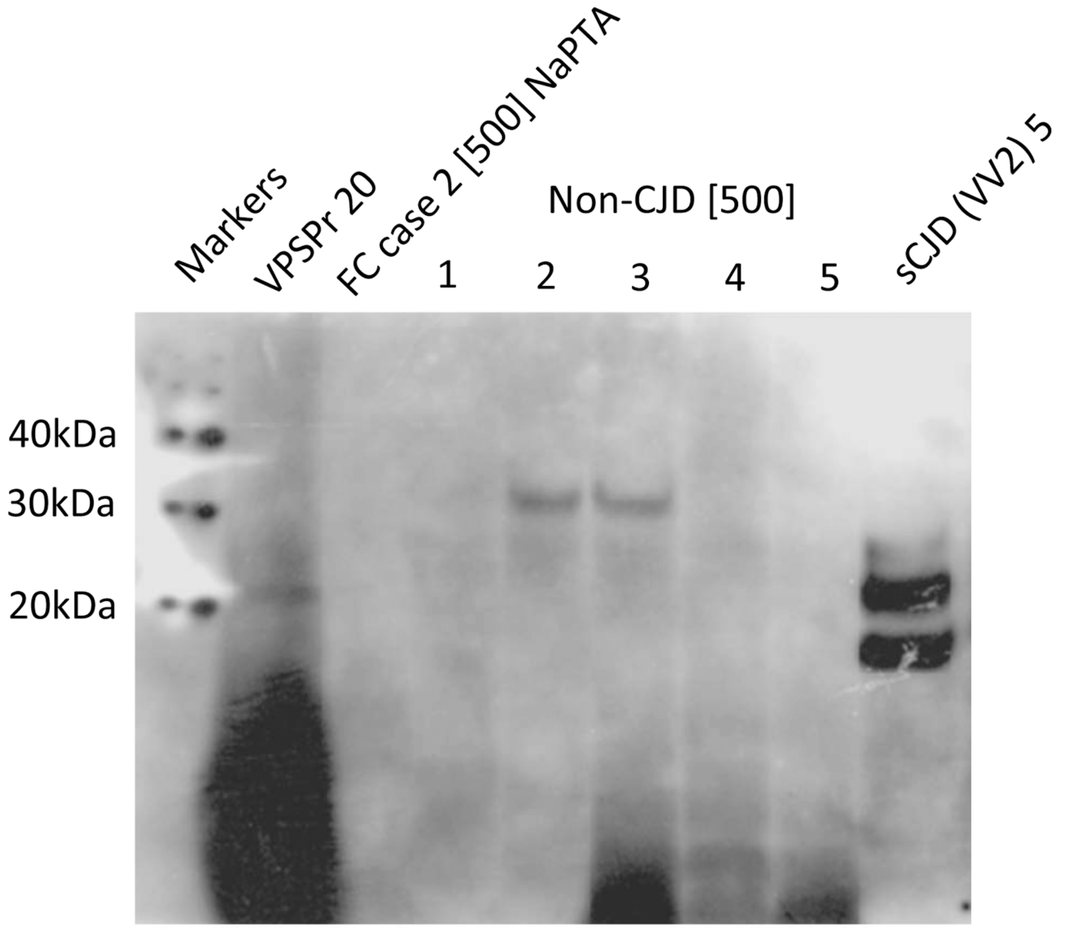

Supplementary figure 5.

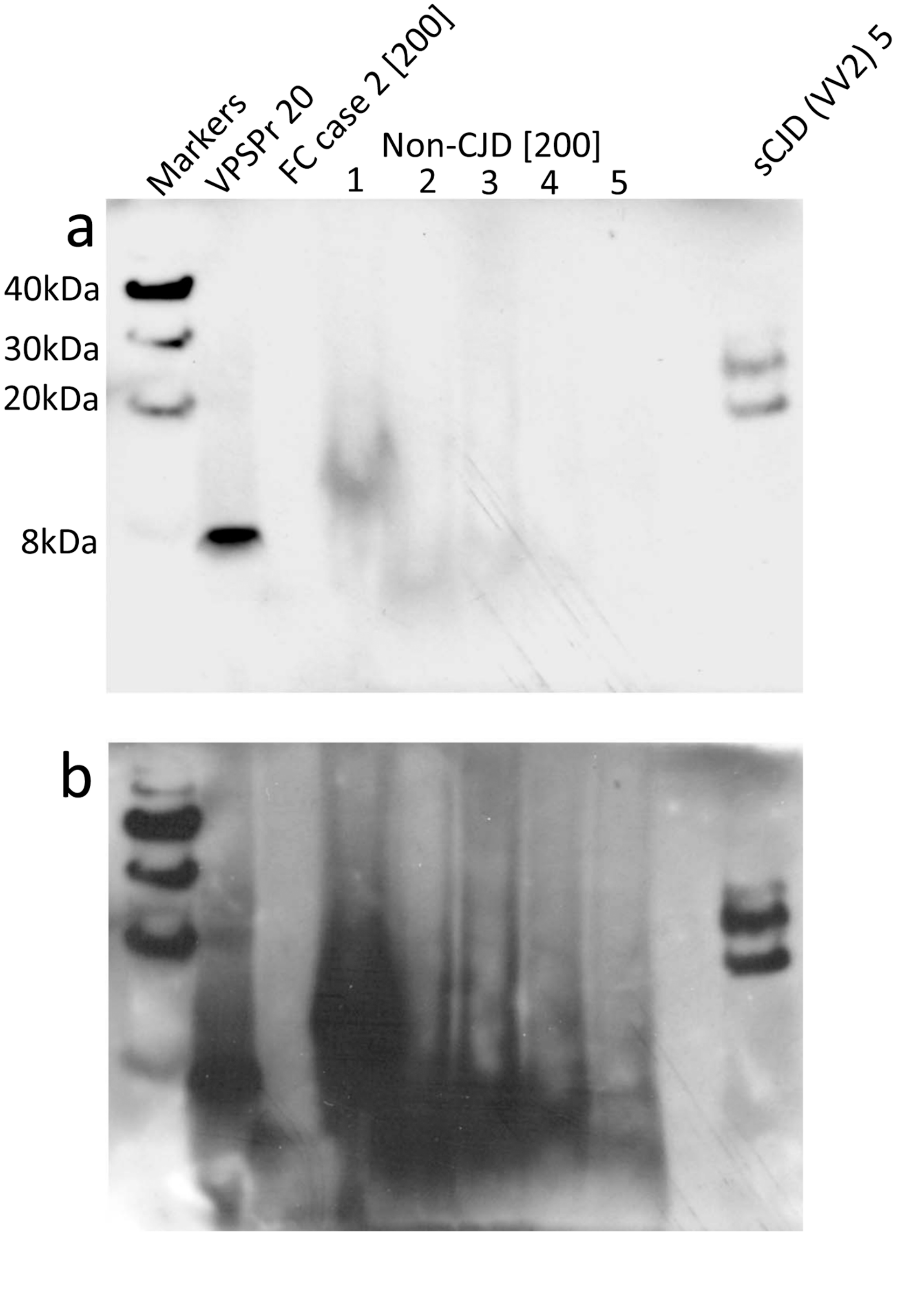

Supplementary figure 6.

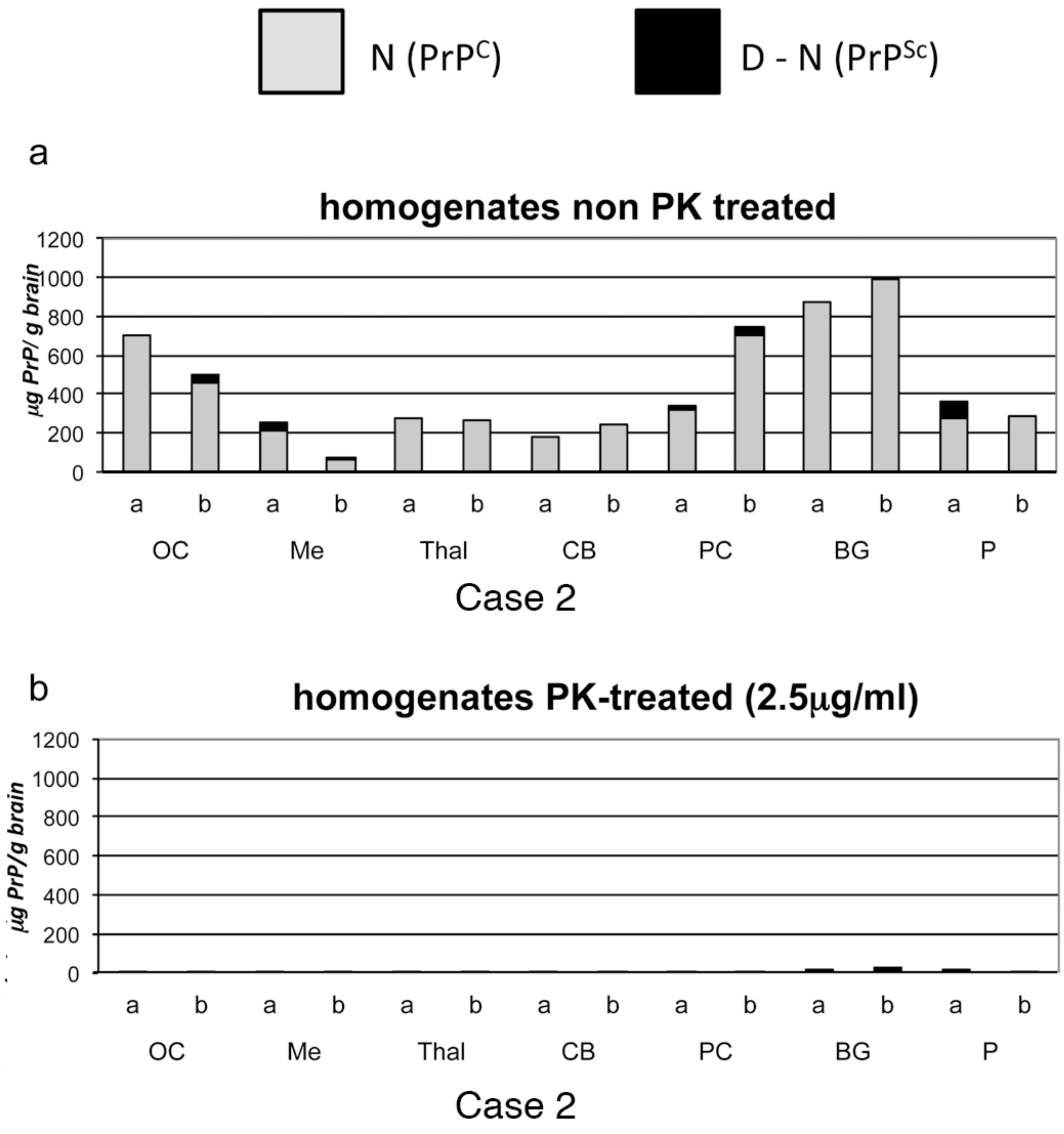

Supplement: Additional file 1: Figure S1 — a. Cranial MRI image (Case 3). b. Cranial MRI images (Case 4). c. EEG (Case 4). d. Cranial MRI (case 6). e. DWI images (Case 6). Figure S2. PET blot examination following PK digestion of a section from the entorhinal cortex in a case of sFI, and case 2 from the present series. Figure S3. Immunostaining for PrP (antibody12F10) in three cases of FFI reveals focally similar neuritic immunoreactivity. Figure S4. Western blot analysis of PrPres in frontal cortex of the thalamic case 2 compared to cortical samples of known non-CJD neurological control cases, a variably protease sensitive prionopathy (VPSPr) case and a sporadic Creutzfeldt-Jakob disease of the VV2 subtype. Sample loadings (μl of a 10% w/v brain homogenate) are shown for all lanes. Sample loading in square brackets ([]) denote volumes used for NaPTA precipitation prior to PK digestion. Figure S5. Western blot analysis of PrPres in frontal cortex (FC) of the case 2 compared to cortical samples of known non-CJD neurological control cases, a variably protease sensitive prionopathy (VPSPr) case and a sporadic Creutzfeldt-Jakob disease of the VV2 subtype (sCJD (VV2)). Sample loadings (μl of a 10% w/v brain homogenate) are shown for all lanes. Sample loading in square brackets ([]) denote volumes concentrated prior to loading. The positions of the molecular mass of marker proteins (Markers) and the low molecular mass PrPres characteristic of VPSPr are given in kilodaltons (kDa). (a) and (b) show short (3 minutes) and long (30 minutes) exposures of the same Western blot. Figure S6. CDI analysis of brain regions of case 2 after treatment with 0 and 2.5 μg/ml PK. [file 2051-5960-1-72-S1.pdf]
